# Person-centred care in osteoarthritis and inflammatory arthritis: a scoping review of people’s needs outside of healthcare

**DOI:** 10.1186/s12891-021-04190-z

**Published:** 2021-04-09

**Authors:** Jessica L. Fairley, Maheeka Seneviwickrama, Sabrina Yeh, Shane Anthony, Louisa Chou, Flavia M. Cicuttini, Kaye Sullivan, Andrew M. Briggs, Anita E. Wluka

**Affiliations:** 1 Department of Epidemiology and Preventative Medicine, School of Public Health and Preventative Medicine, Monash University, Melbourne, Victoria Australia; 2 Department of Community Medicine, Faculty of Medical Sciences, University of Sri Jayewardenepura, Gangodawila, Nugegoda Sri Lanka; 3 Monash University Library, Monash University, Melbourne, Victoria Australia; 4 Curtin School of Allied Health, Curtin University, Perth, Western Australia

**Keywords:** Arthritis, Osteoarthritis, Person centred care, Rheumatoid arthritis, Patient needs

## Abstract

**Background:**

Arthritis, regardless of cause, has significant physical, social and psychological impacts on patients. We aimed to identify the non-healthcare needs perceived by patients with inflammatory arthritis (IA) and osteoarthritis (OA), and to determine if these differ.

**Methods:**

We electronically searched MEDLINE, PsycINFO, EMBASE and CINAHL (1990–2020) systematically to identify non-healthcare-related needs of people with IA or OA. All citations were screened and quality appraised by two reviewers. Data was extracted by a single reviewer.

**Results:**

The search identified 7853 citations, with 31 studies included (12 for OA, 20 for IA). Six areas of need emerged and these were similar in both group These were: 1) Assistance with activities of daily living especially related to a lack of independence; 2) Social connectedness: need for social participation; 3) Financial security: worry about financial security and increased costs of health-seeking behaviours; 4) Occupational needs: desire to continue work for financial and social reasons, facilitated by flexibility of workplace conditions/environment; 5) Exercise and leisure: including limitation due to pain; 6) Transportation: limitations in ability to drive and take public transport due to mobility concerns. Many areas of need were linked; e.g. loss of employment and requiring support from family was associated with a sense of “failure” and loss of identity, as social isolation.

**Conclusions:**

This review highlights the pervasive impact of arthritis on peoples’ lives, regardless of aetiology, albeit with a limited evidence base. Improved identification and targeting of non-healthcare needs of people with arthritis is likely to improve person-centred care.

**Supplementary Information:**

The online version contains supplementary material available at 10.1186/s12891-021-04190-z.

## Significance and innovations

- People with osteoarthritis and inflammatory arthritis perceive needs for support in many areas of life outside of direct healthcare, related to activities of daily living, exercise, social participation, environment, occupation and transport.
- Despite differences in pathology, the non-healthcare-related needs of people with OA and IA are similar.
- Improved identification and targeting of non-healthcare needs of people with arthritis is likely to improve person-centred care.

## Background

Arthritis affects up to 80% of older adults in developed countries [1], with the two most common forms being osteoarthritis (OA) and rheumatoid arthritis (RA). Osteoarthritis accounts for 2.19% of all years lived with disability (YLDs) for any condition worldwide; while RA accounts for 0.28% of all YLDs [2]. The management of OA is limited with no disease modifying therapy available; the focus of therapy is symptom control and maintenance of function, with joint replacement an option for end-stage disease. For people living with OA, creating a healthcare and broader social environment that supports symptom management and capabilities to effectively self-manage a long-term disease is essential [3]. In contrast, biological therapies have improved morbidity and mortality outcomes in people with inflammatory arthritis (IA), particularly (RA) [4]. With effective treatments available for controlling disease activity, medical practitioners may focus care priorities in this area and other specific health needs (e.g. co-morbidity care), rather than broader health and social needs that may be relevant to the person. Notably, in people with other rheumatologic conditions, including lower back pain, addressing needs outside of direct medical care to provide holistic care, can improve quality of life [5].

While disease management may necessarily vary between individuals, all form of arthritis are associated with common impacts on people’s lives, in particular a loss of function, loss of dexterity, mental health sequelae and limitations in participation, often leading to unfavourable social consequences [6–8]. While these domains of impact are common, the nature of the impacts will vary between by disease. For example, those with inflammatory arthritis taking immunosuppressant medication may have specific occupational- and transport-related needs, to avoid situations that increase their risk of infection which are not relevant to those with osteoarthritis. To optimise holistic care, it is necessary to understand peoples’ non-healthcare related needs, beyond direct healthcare provision, such as the social determinants of health [9]. Accordingly, we aimed to identify current knowledge regarding the non-healthcare needs perceived by patients with OA and IA, and examine differences where identified.

## Methods

A systematic scoping review was performed to provide an overview of the literature around the patient perceived non-healthcare needs of people with OA and IA, in line with the PRISMA guidelines for scoping reviews [10]. This was conducted within a larger project examining patients’ perceived needs relating to musculoskeletal health [11].

### Search strategy

Medline, EMBASE, CINAHL and PsycINFO were electronically searched (1990 to September 2020) using a combination of keywords and MeSH terms related to perceived non-healthcare needs in people with OA and IA separately. A comprehensive search strategy was co-developed iteratively by a multidisciplinary team involving an academic librarian, input from a patient representative and four clinician researchers (Rheumatologists, Physiotherapist and public health physician) (Supplement Material Figures S1a, S1b, S2a, S2b, S3a, S3b, S4a, S4b).

### Inclusion and exclusion criteria

English-language studies were included examining people older than 18 years with OA and IA. IA was defined as any joint disease where the primary mechanism was inflammation or synovitis, including RA, systemic lupus erythematosus (SLE) and psoriatic arthritis (PsA), excluding OA or crystal arthritis. Regarding OA, both clinical and radiological definitions were included. Studies had to report on perceived non-healthcare needs relevant to OA or IA. The concept of a “need” is complex, and currently without a consensus definition in the literature [12]. Broadly, we defined “need” in the context of health as a person’s desires, expectations and requirements [12], aligned with other work in this space [11, 13]. Non-healthcare services were defined as interventions, supports or structures not directly relating to healthcare, to support and assist people with functional limitation from their disease. Full text articles were included; no restrictions on study design were made to ensure a broad focus was maintained.

### Study selection

Each title and abstract was screened for eligibility independently by 2 investigators (MS, SY, LC or JF). Full texts of studies meeting the inclusion criteria were retrieved and assessed (MS, LC or JF). Discrepancies were reviewed with an additional investigator (AW) to reach consensus.

### Data extraction and analysis

Data were extracted by one author (MS, LC or JF) using a standardised data extraction form designed to capture demographic data, aims and description of study methods and outcomes. Included studies were reviewed by one author (MS, JF or AW) to identify aspects of non-healthcare needs, using principles of meta-ethnography to synthesise qualitative data [14]; the most common analytic approach for synthesising qualitative data from primary studies [15]. The principle of meta-ethnography is to empirically derive, though an inductive analytic frame, new concepts, interpretations, or theories that extend or go beyond findings of any individual study. In this way, a body of qualitative evidence contributes to the development of new themes or concepts [16]. The analytic approach used in this review followed the seven phases described by Noblit and Hare [17] and more recently described by France et al. [18]. Initially, one author (MS) reviewed each study in detail, reading the text several times to inductively derive an overarching framework of concepts and underlying themes from the yield of the qualitative studies. This framework was directly informed by data extracted from the primary studies and any pertinent points raised by the authors of the primary studies in the discussion, as recommended by France et al. [18]. In developing the framework of themes and subthemes each primary study was compared to the others to identify comparability, similarity or opposition based on grouping concepts. Once primary studies were translated into each other two senior rheumatologists (FC, AW) independently reviewed the framework of concepts and themes to ensure clinical meaningfulness and face validity.

Quantitative meta-analysis was not possible due to nature and heterogeneity of included studies. For this reason, quantitative data were reported narratively.

### Risk of Bias assessments

Risk of bias assessments were performed independently by 2 investigators (JF, SA, AW or SY). Discrepancies were reviewed by a third investigator (AW) to reach consensus (Supplementary Material Table S1, S2). For qualitative studies, the Critical Appraisal Skills Programme (CASP) tool was used to assign risk of bias estimates [19]. For quantitative articles, the method described by Hoy et al. (Supplementary Material Appendix S1) was used to assess internal and external validity of studies [20]. Studies were deemed to be at low risk of bias if scoring eight or more “yes” answers, at moderate risk if scoring six to seven, and at high risk of bias when scoring less than six [20]. For qualitative studies, the Critical Appraisal Skills Programme (CASP) tool was used to assign risk of bias estimates [10].

## Results

**Fig. 1:**
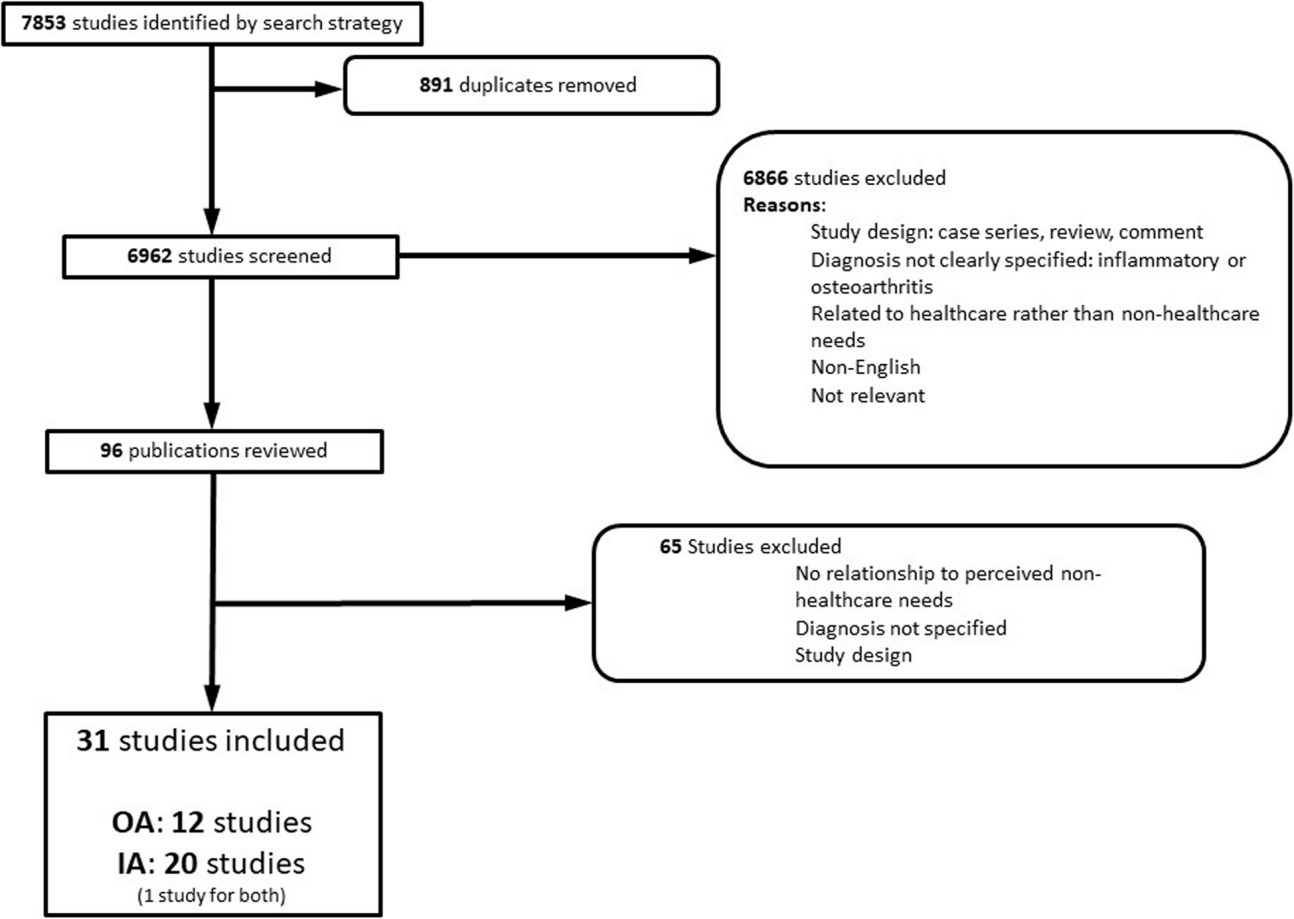
PRISMA Diagram

The search strategy identified 7853 potentially relevant abstracts, of which 891 were excluded as duplicates, and 6962 excluded after abstract screening (Fig. 1: PRISMA Diagram). Ninety-six manuscripts were retrieved for review, with 31 included in the final review. Twelve (39%) studies related to OA, and 20 (65%) related to IA, with one study involving participants with OA and IA with outcomes reported separately. Description of the included studies is presented in Table 1.

**Table 1:** Included manuscripts relating to scoping review of consumer perceived other service needs related to osteoarthritis and inflammatory arthritis, including rheumatoid arthritis and other inflammatory conditions

**Osteoarthritis**						
**Author (year)** **Country**	**Diagnosis**	**No. of subjects**	**Source of participants**	**Age & Gender**	**Aim**	**Study type/design**
Ackerman (2013) [21] Australia (High income^a^)	ACR criteria or radiology reports	*N* = 126 Hip OA: 31% Knee OA: 63% Hip + Knee OA: 6%	Rheumatology or Orthopaedic outpatient clinic (mixed public and private hospitals)	Age(median): 67 years (IQR 57–73) Female 60%	To understand barriers to participation in community-based arthritis self-management programs and patient preferences for self-management education.	Quantitative Questionnaire
Al-Taiar (2013) [22] Kuwait (High income^a^)	Clinical	*N* = 39 Knee OA	Waiting list for TKJR for severe knee OA in the only public orthopaedic hospital in Kuwait	Age (mean): 62.5 +/− 7.9 years Female 100%	To explore the pain experience and mobility limitations as well as the patient’s decision making process to undertake knee joint replacement	Qualitative, focus group discussions
Baumann (2007) [23] France (High income^a^)	Diagnosis methods not specified	*N* = 96 Knee OA 66% Finger OA 50% Hip OA 46%	Customers of 10 pharmacies in 10 towns in 10 regions randomly selected from 22 French regions. The first 10 customers who came to purchase any medication.	Age(mean): 65 years (range 42–89 years) Female 81%	To understand the expectations of patients with OA to use these to improve healthcare provision and the doctor-patient relationship	Qualitative Focus groups
Bukhave & Huniche (2014) [24] Denmark (High income^a^)	Clinical	*N* = 31 Hand OA	Referred by a doctor or volunteers (via an article in Danish Rheumatism Association magazine)	Age(mean): 62.9 years (range 38–89 years) Female 84%	To explore perspectives on activities and participation in everyday life among people with hand OA	Qualitative, semi-structured interviews
Chan (2011) [25] Hong Kong (High income^a^)	Clinical (ACR Criteria)	*N* = 20 Knee OA	GP clinic	Age(mean): 57.05 +/− SD 10.79 years Female 65%	To evaluate the influence of pain patterns on quality of life, and to investigate interpretation and coping strategies	Qualitative, semi-structured interviews
Ilori (2016) [26] Nigeria (Lower-Middle Income^a^)	Clinical (ACR Criteria)	*N* = 270 Knee OA	GP clinic	Age: NR Gender: NR	To assess family and social supports, and health impact on patients with knee OA	Quantitative Questionnaires
Hill (2010) [27] United Kingdom (High income^a^)	Clinical (by GP or rheumatologist)	*N* = 29 Hand OA	GP or rheumatology outpatient clinic	Age(mean): primary 62.4 years, secondary 63.6 years Female: primary 80%, secondary 93%	To investigate the functional impact of hand OA on everyday life	Qualitative: Semi-structured interviews
Kao (2014) [28] Taiwan (High income^a^)	Stage 1 or 2 knee OA (Ahlback)	*N* = 17 Knee OA	Orthopaedic outpatient clinic (2 hospitals)	Age (mean): 49.6 +/− SD 4.2 years (range 43–55 years) Female 82%	To understand the illness experiences of middle-aged adults with early knee OA	Qualitative, semi-structured interviews
Kjeken (2013) [29] Norway (High income^a^)	Clinical (ACR Criteria)	*N* = 125 Hand OA	Rheumatology and orthopaedic outpatient clinics (public hospital)	Age(mean): 64.5 years Female 98%	To explore self-management strategies in hand OA, especially strategies for daily activities	Qualitative and quantitative Questionnaires
Leung et al. (2019) Singapore (High Income^a^) [30]	Clinical (ACR Criteria)	*N* = 45 Hand OA	Rheumatology outpatient clinic (dedicated hand OA clinic)	Age (mean): 64.3 years (range 51–82 years) Female 91.1%	To explore patients’ perspectives in priorities for core domains for clinical trials related to hand OA.	Quantitative Questionnaires
Neville (1999) [31] Canada (High income^a^)	Clinical	*N* = 197 • RA: 57 • SLE: 27 • OA: 41 • Back Pain: 55 • Systemic sclerosis: 17	Rheumatology outpatient clinic (public or private, multicentre)	Age(mean): 60 +/− 15 years Female83.2%	To identify concerns & learning interests of arthritis patients	Quantitative Descriptive cross-sectional self-administered questionnaires
Tanimura (2011) [32] Japan (high income^a^)	Clinical	*N* = 362 Knee OA	Orthopaedic outpatient clinics (predominantly public hospitals)	Age (mean): 72.4 +/− 9.6 years Female 281/362 (78%)	To develop an instrument to assess difficulties in daily life of patients with knee OA, and to investigate factors influencing difficulties in life	Quantitative Questionnaires
**Inflammatory arthritis**						
**Author (year)** **Country**	**Diagnosis**	**No. of participants**	**Source of participants**	**Age & Gender**	**Primary study aim**	**Study type/ design**
Alten (2019) [33] Europe/ Canada (High income^a^)	Clinical	*N* = 1231 RA	Online advertising, previous survey participants	Age: most common 40–59 years (48%), 30% > 60 years, 22% < 40 years Female 58%	To understand the impact of RA on patients’ lives.	Quantitative Questionnaires
Been-Dahmen (2017) [34] The Netherlands (High income^a^)	Not specified	*N* = 20 • RA: 16 • PsA: 2 • AS: 2	Rheumatology outpatient department	Age: most common 55–64 years (10/20; 50%), 5/20 < 55 and 5/20 65+ years. Female 14/20 (70%)	To identify support needs of outpatients with rheumatic disorders and preferences for provision of self-management support	Qualitative: Face-to-face interviews 6/20 Focus Group interviews 14/20
Bergsten (2011) [35] Sweden (High income^a^)	Clinical	*N* = 16 RA	Rheumatology hospital; outpatient clinic or rehabilitation service	Age (mean): Women 62 years (Range 28–82 years), Men 61 years (range 42–70 years) Female 10/16 (62.5%)	To generate a model for how patients manage RA in everyday life	Qualitative Face-to-face interviews
Carter (2019) [36] Australia/ New Zealand (High income^a^)	Clinical	*N* = 21 PsA with foot involvement	Rheumatology outpatient department	Age (mean): 53 +/− 13 years Female 62%	To explore how foot problems impact on the lives of people with PsA.	Qualitative: semi-structured interviews
Cunha-Miranda (2010) [37] Portugal(High income^a^)	ACR Criteria	*N* = 233 • RA	Rheumatology outpatient department	Age (mean): 55.13 +/− 14.49 years Female 82.5%	To determine principle sources of disease information in RA patients, unmet needs and patient involvement in decision making.	Quantitative: Questionnaires
Giacomelli (2015) [38] Italy (High income^a^)	Not specified	*N* = 743 • RA:327 • PsA:214 • AS:200	Rheumatology outpatient department	Age: 493 patients > 45 years of age Female 58%	To patient involvement in medical decisions, quality of life and unmet needs after introducing biological therapies	Quantitative: Questionnaires
Hamnes (2011) [39] Norway (High income^a^)	Clinical (GP or specialist) ACR criteria	*N* = 16 • RA: 8 • Fibromyalgia (FM): 8	Patients awaiting self-management programmes (SMP)	Age (mean): 51.4 years Female 13/16 (81.2%)	To identify expectations prior to a one-week self-management program, and outcomes	Qualitative: Semi-structured interviews
Henchoz (2013) [40] Switzerland (High income^a^)	Clinical (Rheumatologist) ACR functional classes I-III	*N* = 89 All RA	Rheumatology outpatient clinic (tertiary centre)	Age (mean): 58.4 years Women 71/89 (79.8%)	To examine patients’ perceptions of exercise benefits, barriers, and their preferences for exercise	Quantitative: cross sectional study, using self-administered questionnaire
Herrera-Saray (2013) [41] Mexico & Colombia (Both Upper-Middle-Income^a^)	Disabled users of assistive devices Not mentioned	*N* = 15 Inflammatory Arthritis 9/15 • RA: 4 • Spondylo-arthropathy: 5 • Amputee (any cause): 6	Rheumatologist & snow-ball method	Age (mean): 41 years Women 6/15 (40%)	To identify usage/accessibility problems faced by the disabled and users of assistive devices, and physical barriers that limit their mobility	Qualitative: In-depth interviews
Kostova (2014) [42] Switzerland (High income^a^)	Clinical	*N* = 20 All RA	Patients selected by rheumatologists as successful in dealing with implications of RA	Age: Women 13/20 (65%)	To investigation the relationship between social support and acceptance in patients with RA	Qualitative Semi-structured interviews
Kristiansen (2012) [43] Denmark (High income^a^)	Clinical	*N* = 32 All RA	Outpatient clinics (Multicentre)	Age (mean): 58.2 years Women 19/32 (59.4%)	To explore effects of RA on everyday life	Qualitative Focus groups
Laidmae (2009) [44] Estonia (High income^a^)	Clinical	*N* = 808 All RA	Hospitals and health centres (multicentre)	Age: 66% (*n* = 533) over 56 years [No range or mean age given] Female: 687 (85%)	To investigate the impact of RA on quality of life, and the role of support and assistance from family members/acquaintances	Quantitative: cross-sectional study, using self-administered questionnaire
Lempp (2006) [45] England (High income^a^)	Clinical	*N* = 26 All RA	Rheumatology outpatient clinics (multicentre)	Age(mean): 56 years Female 22/26(84.6%)	To understand personal experiences of living with RA, and impact of RA upon patients’ lives	Qualitative Semi-structured interviews
Neville (1999) [31] Canada (High income^a^)	Clinical	*N* = 197 • RA: 57 • SLE: 27 • OA: 41 • Back Pain: 55 • Systemic sclerosis: 17	Rheumatology outpatient clinic (public and private, multicentre)	Age (mean): 60 +/− 15 years Female 164/197 (83.2%)	To identify concerns & learning interests of arthritis patients	Quantitative Descriptive cross-sectional self-administered questionnaires
Sato (2008) [46] Japan (High income^a^)	Clinical	*N* = 364 All RA	Commercial healthcare database services (patient records)	Age (mean): 45.5 +/− 8.4 years Female 288/364 (79.1%)	To describe the nature of benefit finding in rheumatoid arthritis including predictive social factors and impact on mental health	Quantitative Questionnaires
Strand (2015) [47] USA/Europe (high income^a^)	Clinical	*N* = 1958 All RA	Internet survey	Age (mean): 46 +/− 10.4 years Female 100%	To identify effects of RA and the impact of goal-setting strategies	Quantitative Questionnaires (2 different surveys)
Sverker (2015) [48] Sweden (High income^a^)	Clinical (ACR criteria)	*N* = 48 All RA	Rheumatology outpatient clinics	Age 20–45: *n* = 9 Age 46–55: *n* = 11 Age 56–60 *n* = 18 Age 61–63: *n* = 10 Female 71%	To explore the experience of early RA in everyday life	Qualitative Semi-structured interviews
Thomas (2019) [49] UK (High income^a^)	Clinical	*N* = 15	Rheumatology outpatient clinic	Age (mean): 56 years (range 29–80 years) Female: 12/15 (80%)	To explore the perspectives, experiences and strategies employed by people with RA who engage in regular physical activity.	Qualitative Semi-structured interviews
Van der Meer (2011) [50] Netherlands (High income^a^)	Clinical	*N* = 14 All RA	Rheumatology outpatient clinic	Age (mean): 47 +/−2.9 years Female 12/14 (85.7%)	To investigate patient experiences and needs in work participation of people with RA treated with anti-tumour necrosis factor (TNF) therapy	Qualitative In-depth interviews
Wollenhaupt (2013) [51] Germany (High income^a^)	Not specified	*N* = 318 All RA	Members of a German association for rheumatic diseases	Age: 60+ 63.5% Female 83.3%	To assess quality of life as well as perceived needs and expectations for treatment and support	Quantitative Questionnaires

^a^Income stratification according to World Bank Country and Lending Groups 2019 Fiscal report based on Gross National Income per Capita [52]

Twelve studies investigated the perceived non-healthcare needs of people with OA [21–32]. Included studies were all from high income countries except one (Nigeria [26]), involving 17–362 participants. Mean age ranged from 49.6 to 72.4 years, with studies including predominantly women (60–100% female). Participants were recruited from the ambulatory care settings in all studies; usually outpatient clinics (either rheumatology or orthopaedic [22, 28, 30, 31] or primary care [25].

Five studies included people with knee OA [22, 25, 26, 28, 32], four hand OA [24, 27, 29, 30], two a mixture of joint involvement [21, 23] and one did not specify [31]. OA was usually diagnosed clinically [21, 22, 24–27, 29–32]. OA was mild in two studies [28, 30] and end-stage in another [22] but most commonly severity was unspecified (9/12, 75%). Six studies used qualitative methods (semi-structured interviews [24, 25, 27, 28] or focus group discussions [22, 23], five quantitative methods [21, 26, 30–32], and one both [29]. Of qualitative studies, five were assessed as low risk of bias [22, 24, 25, 27, 28] and one moderate risk of bias [23] (Supplementary Material Table S1). Of quantitative studies, five were assessed as moderate risk of bias [21, 26, 29, 30, 32], and one at high risk of bias [31] (Supplementary Material Table S2).

Twenty studies discussed non-healthcare needs for IA [31, 33–51]. All but one study included predominantly female participants (58–100%) [41], with all studies from high-income countries. Included participants were generally over 50 years of age [31, 33–40, 42–45, 48, 49, 51], although in four studies mean age was in the 40s [41, 46, 47, 50]. IA was usually diagnosed clinically [31, 33, 35–37, 39, 40, 42–50]. RA was the most common diagnosis [34, 35, 37–40, 42–48, 50, 51] [33, 49], with participants usually recruited from outpatient clinics [31, 35, 36, 38, 40, 41, 43–45, 48–50]. Nine quantitative studies used questionnaires [31, 33, 37, 38, 40, 44, 46, 47, 51], while 11 qualitative studies used interviews [34–36, 39, 41, 42, 45, 48–50] or focus groups [43]. Of the qualitative studies, six were assessed as low risk of bias [34, 42, 45, 48–50] and five at moderate risk of bias [35, 36, 39, 41, 43] (Supplementary Material Table S1). Of the quantitative studies, one was assessed as low risk of bias [38], two at moderate risk of bias [44, 46] and six at high risk of bias [31, 33, 37, 40, 47, 51] (Supplementary Material Table S2).

### Areas of need identified

This review identified six key areas of need in people with IA or OA, common to both groups (Table 2, Table 3).

1. Activities of daily living (ADLs) both inside and outside the home

**Table 2:** Results of scoping review of consumer perceived other service needs related to osteoarthritis

Author, Year	Results
***ACTIVITIES OF LIVING BOTH INSIDE AND OUTSIDE THE HOME***	
Ackerman (2013) [21]	• Cannot get out of house without assistance • Difficulties walking due to OA, limited mobility
Al-Taiar (2013) [22]	• Inability to do household chores, mobility limitation • Many participants have domestic helpers • “Failure” to fulfil obligation to take care of the family despite their pain/mobility limitation; feeling helpless/less valuable
Bergsten (2011) [35]	• Need for support from family and friends when doing household duties, personal care or everyday activities • Struggling to accept help from others; wanting to do more
Bukhave & Huniche (2014) [24]	• Difficulty handling small objects e.g. cutlery, glasses, gadgets, chargers, plugs and devices for connecting gadgets to power supplies, computers, cell phones (especially if buttons too small), money and payment systems (credit cards easier) • Difficulties determined by design/operation of the actual gadget • Dependency on help from others (partners most important providers of support), particularly with respect to the performing of household chores and self-care (grooming, hair dryers, buttons, tying shoes) • Singles with small networks experienced huge challenges • Need for external help at time; expensive • Importance of good grooming to participants • Special equipment, assistive devices or orthoses can improve performance; e.g. self-adapted knife
Chan (2011) [25]	• Reliant on support from the family or paid supports • Need to be accompanied by others when going out
Hill (2010) [27]	• Limited function in day-to-day activities including self-care activities 66% (cutting fingernails, drying after showering, toileting) • Difficulties with opening packaging, peeling fruit and vegetables, cutting • Limitation of hobbies/past times • Gender differences: men reported difficulties with manual work and particular hobbies (fishing, car mechanics), women reported difficulties with home-making tasks (housework, cooking) • Feelings of “frustration” at inability to do things in 55%; may lead to depression • Transitioning from normal function “taking it for granted”; loss of identify/sense of self because of being unable to do things previously done • Inability to conform to social norms due to functional constraints causing embarrassment/self-consciousness • Utility of assistive devices/adaptations to improve function and independence
Kjeken (2013) [29]	• Strategies to improve function in daily activities: • Assistive devices: opening packaging, cutting food • Adapting tools/materials/working techniques: e.g. facilitating lifting/carrying, housework, opening packaging • Practice activity pacing: planning daily activities and rest breaks to enable task completion • Stop or avoiding certain activities • Importance of positive thinking in completing tasks: focussing on what you can do, not pain or limitation, perseverance • Communication: ask/apply/pay for help, telling people about needs/problems
Neville (1999) [31]	• 75% report needing more help carrying out daily tasks
Tanimura (2011) [32]	• Restriction of daily activities 70.5% • Taking more time to complete daily activities 66% • Difficulty sitting on traditional straw matting (“tatami”) 94.7%, sitting up/squatting down 93.7%, going up or downstairs 61.2%, sitting in same position for extended periods 93.1%, carrying heavy objects (88.4%)
***SOCIAL PARTICIPATION NEEDS***	
Al-Taiar, (2013) [22]	• Mobility restriction affects social life (including attending events like weddings) • Whole family affected rather than leave participant at home alone; especially young children/teenagers requiring supervision
Baumann et al., (2007) [23]	• Emotional distress as well as physical limitations; difficulty communicating struggle with family or doctors • Unrecognised disability; lack of recognition by family and friends (not seeing OA as a “real” disease), community (e.g. access to disability permits), lack of OA-related research and media coverage • Importance of support from others with the same condition; “It’s so nice to feel you are not the only one suffering”
Bukhave & Huniche, (2014) [24]	• Limited participation in activities requiring withdrawal from group activities (e.g. skiing, canoeing, dancing, woodwork and holding dinner parties) resulting in reduction of social network • Difficulty caring for grandchildren, including lifting and carrying children
Chan, (2011) [25]	• May have to cut down or abstain from social activities • Often limited choice of social activities depending on available transportation and walking distance • Difficulty playing with/looking after grandchildren
Ilori (2016) [26]	• Social support most commonly provided by children (68.8%) • Perceptions of “good health” significantly more common in those with strong support from family (69.9%) and friends (71.6%) cf. those with weak support from family (47.1%) or friends (59.6%) • High functional health significantly more common in those with strong support from family, friends and significant other than those with weak support.
Hill (2010) [27]	• Unable to conform to social norms due to functional constraints, causing feelings of embarrassment • Comparison with others made people more aware of disability, but sometimes reminded people that others were worse off
Leung et al. (2019) [30]	• Hand OA had significant impact on ability to participate in social roles in 33.3%, emotional health and mood in 28.9%, ability to participate in social ability in 31.1% and appearance of hands/self-image in 37.8%
Neville (1999) [31]	• 20% of OA patients interested in a self-help group
Tanimura (2011) [32]	• Lack of recognition of knee pain by others 58.2%
***FINANCIAL NEEDS AND SECURITY***	
Chan (2011) [25]	• Monetary costs of treatments affect health seeking behaviour
Hill (2010) [27]	• 2/29 forced to retire from work due to hand problems; significant financial implications of giving up work • Struggling to handle money and write cheques due to hand OA
Kao (2014) [28]	• Reduction of work affecting household income (87.5% labourers); 61.5% were the main income earner
***OCCUPATIONAL NEEDS***	
Bukhave & Huniche (2014) [24]	• Struggle to keep working until retirement age • Some had option for flexibility in arrangements with employers; depends on individual work demands, may need to change to a job where demands match hand function • Often lack of adaptation of work environment and technical aids, and lack of knowledge concerning workplace adaptations and technical aids that could have been offered by the employer • Flexibility important
Chan (2011) [25]	• Impacts on work life included: tiring easily, feeling inconvenient, less efficient, need to take sick leave, need to quit job, fewer business trips / do less business • Some forced to change job/resign/early retirement
Kao (2014) [28]	• Need to reduce work, adjust work content and exchange work • Limitation of work due to pain
Leung et al. (2019) [30]	• Hand OA had a significant impact on work productivity in 33.3%
***EXERCISE AND LEISURE-RELATED NEEDS***	
Al-Taiar (2013) [22]	• Restriction of leisure activities of the whole family due to patient’s disability
Bukhave & Huniche (2014) [24]	• Need to change/avoid exercise, replacing lost activities with more manageable ones e.g. aqua gymnastics • More sedentary/passive activities (e.g. watching TV); difficulties with many activities e.g. golf, skiing, canoeing, fishing, bicycling, gardening, knitting, sewing, and holding books while reading
Chan (2011) [25]	• Inability to do exercise a major concern; some needed to give up recreational/social activities altogether
Kao (2014) [28]	• Exercise limitation due to pain • Need to choose mode of exercise carefully and change to different activities
Tanimura (2011) [32]	• Incapable of pursuing hobbies/challenges 68.8% • Incapable of attending local activities 80.4%
***TRANSPORT NEEDS AND ENVIRONMENTAL MODIFICATION***	
Ackerman et al. (2013) [21]	• Transport difficulties in 22%
Bukhave & Huniche (2014) [24]	• Difficulty with handling the shift, holding on to the steering wheel, opening doors and the boot and handling the petrol cap of a car • Difficulty riding a bike e.g. hand brakes, shifting gears, lamps and locks • Difficulty with public transport e.g. holding on to straps or poles during exacerbating pain/other symptoms
Chan (2011) [25]	• Difficulty going out, particularly taking public transport; worsens with disease progression • Lack of suitable public transport facilities • Use of walking sticks
Kao (2014) [28]	• Did not enjoy travelling, especially getting in and out of the car • Pain an inconvenience e.g. climbing stairs, needing to look for seated toilets • Need to use analgesia prior to outings

**Table 3:** Results of scoping review of consumer perceived other service needs related to inflammatory arthritis

Author, Year	Results
***ACTIVITIES OF LIVING BOTH INSIDE AND OUTSIDE THE HOME***	
Alten (2019) [33]	• 23% of RA patients found personal grooming difficult due to pain and fatigue • Inability to complete activities made people feel anxious, frustrated or “like a failure”, especially in patients > 40 years old
Been-Dahmen (2017) [34]	• “Nothing is as difficult as changing your lifestyle” • Extent of support required determined by disease stage, presence of symptoms and change in situation • Patients struggle to accept help; less ready to accept help from children than partners
Carter (2019) [36]	• Change in routine due to foot pain in PsA with needing to stop/modify activities (cleaning, shopping, cooking, gardening) • Difficulty with foot care
Cunha-Miranda (2010) [37]	• 32.3% report impact of RA on quality of life; 26.4% said RA made life less enjoyable; symptoms of RA controlled daily lives in 25.1% • 31.8% difficulty performing ADLs • 25.1% constantly tired • Difficult tasks included gardening, sports, household chores, sleeping
Hamnes (2011) [39]	• “Now I have to ask for an increasing amount of help and that transition is difficult” • Provision of techniques and aids that could make work and daily activities easier
Herrera-Saray (2013) [23]	• Amputees found to have greater independence than patients with rheumatic disease • May “get used to” new circumstances
Kostova (2014) [42]	• Family are most important source of support, esp. spouses and children, strong motivation to avoid becoming “passive” victim of disease and a vital source of emotional and practical support • Loss of identity because unable to do housework as previous • Difficulty with asking for help; more likely to accept help if offered spontaneously/needs anticipated rather than having to ask
Kristiansen (2012) [43]	• Need to set up personal and practical support in the household
Laidmae (2009) [44]	• Continuous vs. occasional support at home; 29% living alone
Lempp (2006) [45]	• Required practical help from family members for activities of daily living • Children became caregivers
Neville (1999) [31]	• 93% RA patients need help to carry out daily tasks
Sato (2008) [46]	• Difficulties at home due to RA in 18%
Strand (2015) [47]	• 60% difficult to perform “normal” activities due to RA; worrying about losing independence 75% • Difficulty making plans due to pain, mobility restriction and fatigue • Difficulty with housework (39%), sleeping (28%), shopping (24%), cooking (16%)
Sverker (2015) [48]	• Difficulties with self-care such as dressing, doing housework, gardening and shopping • Difficulties were due to pain and stiffness, and functional limitations from deformities.
Wollenhaupt (2013) [51]	• Impact of RA on life rated as “rather bad” or “very bad” • Housework requiring “a lot of effort” for 23.6%; 5.2% unable to do housework, especially running errands/shopping (restriction in lifting/carrying shopping bags in 57.7%) • 60% of respondents “more or less” dependent on a third-party in day-to-day activities, usually upon partner or family/friends
***SOCIAL PARTICIPATION NEEDS***	
Alten (2019) [33]	• 35–39% of people reported difficulty with others understanding their disease • Negative impact on relationship with spouse or partner, including sex life and intimacy • Negative impact on inclusion in family and social events • Better understanding from others in those with a partner or children; 43% wished for better understanding of disease impact from others
Been-Dahmen (2017) [34]	• Trusting relationship with professionals, relatives and fellow patients • Emotional support required from relatives; however, they did not always recognise emotional issues. Partners more capable than children. • Most did not need support from fellow patients; some appreciated shared experiences. Most not interested in formal group meetings.
Bergsten (2011) [35]	• Need for support from friends and family, as well as healthcare professionals, but patients need to trust/accept support offered • Need for friends and relatives to understand difficulties faced/problems created by disease
Carter(2019) [36]	• Spending time with family and friends disrupted due to foot symptoms and functional limitations • Lowered mood due to preoccupation with pain; reliance on family members for support • Better understanding/empathy from those with affected family members; some found benefit from support groups • Patients with PsA and foot problems conscious of change to physical appearance and footwear restrictions; demoralised and stigmatised by the appearance of their feet; need to wear clothing and footwear to hide disease; self-conscious and reluctant to use gait aides
Cunha-Miranda (2010) [37]	• 22.4% of RA patients feel “alone” in fighting disease; limited support
Hamnes (2011) [39]	• Shared experiences, support and recognition from peers and validation of problems
Henchoz (2013) [40]	• Community based free physical activity programmes for patients with arthritis
Herrera-Saray (2013) [23]	• Feeling weird/embarrassed among others due to assistive device
Kostova (2014) [42]	• Need for understanding from family members • Lack of visible symptoms meant some family members unable to appreciate patient’s suffering so felt misunderstood
Kristiansen (2012) [43]	• Lack of understanding from friends/wider social environment, withdrawal by patient and their friends • Importance of work in developing social relationships and feeling of belonging • Loss of work leads to loss of social networks • Peer support enables participants to meet others with RA, especially with recent diagnosis, to legitimize personal experiences with symptoms that cannot be objectively measured, role models to show maintaining a close-to-normal life is possible
Laidmae (2009) [44]	• Loneliness & the need to socialize with family & friends; 19% of respondents lonely • 33% of participants living alone (29% of total population are lonely) • Difficult to go out due to financial difficulties, mobility problems and fear of falling a victim of crime • Need for emotional support; emotional support received from the family consists of consolation, encouragement, listening to the worries and providing security
Lempp (2006) [45]	• Retirement leads to loss of social connections • Loss of work means loss of identify, structure of daily life and social life
Neville (1999) [31]	• 44% RA patients interested in self-help groups
Sato (2008) [46]	• Difficulties in personal affairs in 62.9%; sexual difficulties in 14.3% • Emotional support from spouse or partner received by 56.2%; usually parents (27.3%) or children (20.5%)
Strand (2015) [47]	• Isolation in 26%; friends/family not understanding pain and fatigue in 54% • RA affected closest relationships 32% (e.g. playing with children/grandchildren) • More difficult to find a partner 40%, less confident in sex-life 47%, negative affect on intimacy 17%
Sverker (2015) [48]	• Difficulties (due to physical limitation/pain/fatigue) with social relationships, e.g. caring for children/grandchildren, participating in social events and engaging in community life
Wollenhaupt (2013) [51]	• Impact of RA on social activities “strong” to “very strong” 27.6%
***FINANCIAL NEEDS AND SECURITY***	
Laidmae (2009) [44]	• Financial hardship in 60%; restriction of foodstuffs, 20% unable to purchase all medications • Limited sociocultural experiences: cinema/theatre, purchase of books, limited social visits • Suboptimal home environment: absence of warm rooms, hot running water, drainage, opportunity to wash
Neville (1999) [31]	• > 80% patients reported concerns about health care cuts • 72% concerned with future financial coping; 56% concerned with present financial coping
Sato (2008) [46]	• Income protection accessed by 32%; Financial difficulties in 12.9%
***OCCUPATIONAL NEEDS***	
Alten (2019) [33]	• 95% of participants reported leave, retirement or lack of career progression since RA diagnosis; 18% forced to retire and 23% slow career progression • 31% inadequate physical accommodations at work, 36% inadequate emotional accommodations • Barriers to work include difficulty with hand function (44%), pain (43%), unpredictable state of health (34%)
Carter (2019) [36]	• Foot-related disability contributed to loss of work, or difficulty performing jobs due to foot pain and stiffness • Impact of modified footwear on job roles e.g. unable to wear dress shoes or safety boots
Cunha-Miranda (2010) [37]	• RA affected ability to work: 24.7% • Absence from work due to illness: 21.6% (mean duration of absence 16–17 days)
Giacomelli (2015) [38]	• 34% reported difficulties at work; increased work absenteeism in 11, 7.9% retired
Hamnes (2011) [39]	• Need to continue to work, important to avoid disability pension (last resort) • Wanted to know work-related rights and rights related to social security
Kristiansen (2012) [43]	• Need to continue work (with or without special conditions); this helped to maintain ^normal life and sense of normality; need for support to clarify work capacity^ • Work important to social, professional and personal identity, strongly linked to self-esteem • Colleagues as a personal/social network – friends and supports
Laidmae (2009) [44]	• 27% of respondents employed; 25% concerned about losing their job • Perceived job insecurity • Alleviation of financial problems with work
Lempp (2006) [45]	• Flexible working hours; lifts (elevators) at work place- to overcome difficulty in climbing stairs • Desire to continue to work • Loss of work means loss of identity, social network and structure of day
Neville (1999) [31]	• Ability to work and maintain a job
Sato (2008) [46]	• Majority of patients employed; 55–57%; 26% informed work about RA • RA-related difficulties at work in 47.8%; income protection accessed by 32%
Strand (2015) [47]	• Negative impact on work arrangements, productivity and self-confidence • Less productive at work due to RA 71%; less confident at work due to RA 50% • Stop working/retire early 23%, changed type of work 17% or hours 17%; modifications to workstation/environment 12%, pay cut 8% • Regularly > 10 days off work per year in 22% • RA had negatively affected career prospects 9%
Van der Meer (2011) [50]	• Need to improve/increase support in workplace (including from colleagues) • Ergonomic accommodations • Need for control over work; flexible hours and tasks, possibility of working at home, working alone when necessary (to improve concentration) • Easier commuting to work including getting a transfer when travelling a long distance to the workplace, easier parking arrangements • To understand legal work rights: including accommodations at the workplace and concerning disclosure when applying for a job
Wollenhaupt (2013) [51]	• Physical impairment in daily work (inside and outside home) “rather strongly” to “very strongly” impacted in 49.6%
***EXERCISE AND LEISURE-RELATED NEEDS***	
Been-Dahmen (2017) [34]	• Empowered by information about type and necessity of physical exercise, as well as seeing other patients exercising
Bergsten (2011) [35]	• Unable to do particular physical activities
Carter (2019) [36]	• Difficulty with walking especially on uneven ground in those with PsA and foot involvement
Cunha-Miranda (2010) [37]	• Less able to do sports
Henchoz (2013) [40]	• Physical, psychological, functional and social benefits to exercise; arthritis specific barriers e.g. loss of function, pain, stiffness, concern of peers • No programs/consideration for those with arthritis • Non- arthritis specific barriers eg scheduling, cost, lack of time, peers do not exercise, carer responsibilities, etc
Strand (2015) [47]	• Adverse effect of RA on social, family and leisure activities • Limited enjoyable activities (42%) and spontaneity (57%), keeping fit/playing spots (46%), gardening (39%), outdoor activities (33%) • Favourite hobby painful in 31%
Thomas (2019) [49]	• Need for physical activity as a key part of managing RA; symptoms may help to motivate people to be physically active • Options where physical activity also had a social element, as a mode of transportation, dog walking all popular forms of activity • Some hesitation about general group activity classes; concern re: being unable to keep up or lack of understanding of RA
***TRANSPORT NEEDS AND ENVIRONMENTAL MODIFICATION***	
Herrera-Saray (2013) [23]	• Architectural barriers in the home, the workplace and/or outdoors • Lack of design standards for persons with disabilities, e.g. ramps, parking spaces and ample space for movement
Henchoz (2013) [40]	• Environmental modifications favourable for physical activity: availability of facilities free of charge, maintenance of pavements, streetlights
Laidmae, (2009) [44]	• Fear of falling victim of crime (16%); perceived increased risk due to physical impairment and poor health • Transport needs
Strand (2015) [47]	• Difficulty with driving in 17%
Wollenhaupt (2013) [51]	• Unable to drive a car 6.9%

Ten OA studies (Table 2) [21, 22, 24–29, 31, 32] and 15 IA studies (Table 3) [23, 31, 33–37, 39, 42–48] discussed difficulties facing people regarding ADLs.

ADLs of people with OA were limited by symptoms [21, 32], including pain [28]. People with OA worried about needing help from others [21, 22, 24, 25, 28, 31]: 75% in one study [31]. For household chores, help was required either from within the family or external sources [22, 24, 25, 27, 28]. This sometimes contributed to a sense of “failure” to fulfil social obligations e.g. caring for children or partners [22]. People acknowledged that they may struggle to live alone [24]. Cleanliness and grooming were important to people with hand OA [24], who experienced difficulty with fine motor tasks including buttons and lacing shoes [27]. Moreover, hand OA limited the dexterity required for cell phones, cutlery and some payment systems (e.g. coins and notes) [24, 27]. Gadgets with an accessible design and assistive devices improved daily functioning [24, 29].

People with IA frequently received assistance with ADLs [31, 34, 35, 39, 42–45, 48]; up to 93% of people with RA [31]. Partners [42] or children [45] most commonly provided help. Participants disliked accepting assistance from children [34]. Daily activities, particularly housework, were difficult with IA [33, 34, 36, 37, 46, 51]; IA was associated with less enjoyment of life [37]. Pain and stiffness contributed to functional limitations [36, 48]. Functional difficulties could make people feel anxious, frustrated or “like a failure” [33]. Participants often lived alone (29%) [44] and worried about inability to obtain assistance when required [31]. Participants wanted to do more than they could [35]. Compared to amputees, people with IA were less independent and well-adjusted to their circumstances [23]. Participants wanted to tools to make ADLs easier [39], especially environmental modifications. Areas in the home, workplace and outdoors required modifications [23, 40]. Participants valued security and worried about falling victim to crime due to perceived invalidity [44].

2) Social participation needs

Nine OA studies (Table 2) [22–27, 30–32] and 17 IA studies (Table 3) [33–37, 41–44, 46–48, 51, 53] identified issues relating to social participation and connectedness.

Of the OA studies, mobility restriction and lack of suitable transportation significantly limited social participation [24, 25]. Inability to participant in group activities restricted social contact [24], with family activities limited to avoid excluding family members [22]. Hand OA limited participants’ ability to conform to social norms [30] due to functional constraints [27], which could cause embarrassment [27]. Some participants worried about the appearance of their hands [30]. Strong social support was linked to better health perceptions [26]. Caring for grandchildren could be difficult, including limited ability to lift toddlers and change nappies [24, 25]. Both doctors and support networks sometimes failed to recognise OA as a “real” disease, contributing to communication difficulties [23]. People were frustrated by a lack of community support, and underestimation of pain and suffering [32]. Media coverage of, and research into OA was perceived as inadequate [23]. One study suggested that only 20% of people with OA desired access to support groups [31].

In people with IA, social connectedness [31, 35, 39–41, 43, 44, 48, 53] and peer support was critical. Loneliness and withdrawal were common [43, 44], especially for those living alone [44]. IA put strain on personal relationships [46] particularly ability to provide care for others [48], and relationships with partners including sexual function and intimacy [33, 46, 47]. Some experienced lowered mood due to pain [36]. Participants were desperate for support from family and often felt “alone” [37], needing to trust others to accept proffered support [35]. This was exacerbated by limitation of social activities [36, 51] and losing employment [43, 45]. Barriers to social connectedness included perceived lack of understanding [33, 35, 36, 43], financial difficulties, mobility problems and fear of falling victim to crime [44]. Overall, participants felt less able to participate in social events, community life and relationships [33, 36, 48]. Assistive devices made some participants feel embarrassed or “weird” [23]. Participants with PsA and foot involvement felt demoralised or stigmatised by their appearance and need for specialised footwear [36]. Participants were interested in self-help groups [31, 36], which helped with coping and self-management through support, recognition and legitimisation of personal experiences and problems [39, 43]. These groups facilitated participants building new relationships [39, 43], and proving that normality is possible [43]. Having relatives with the same condition helped with coping [36]. Peer groups supporting physical activity were desirable [40].

3) Financial needs and security

Three OA studies (Table 2) [25, 27, 28] and three IA studies (Table 3) [31, 44, 46] investigated financial needs and security.

OA studies focussed on the cost of health-seeking behaviour [25] and need for financial security. Work capacity was limited by disease [27, 28].

In IA*,* financial stress was common [31, 44]. Income protection was accessed by 32% [46]. Participants from Estonia described financial concerns limiting their access to basic needs including food, running water and heating, as well as sociocultural experiences [44].

4) Occupational needs

Four OA articles (Table 2) [24, 25, 28, 30] and 13 IA studies (Table 3) [31, 33, 36, 38, 39, 43–47, 50, 51] identified needs related to work.

People with OA wanted a flexible workplace [24, 25, 28] to facilitate work retention [24]. Flexibility entailed the need for regular breaks and environmental modification, although aides and environmental modifications were often not available [24]. Failure of these supports could require people to change employment [24, 25, 28]. Hand OA could limit work productivity [30].

IA often affected ability to work, particularly productivity, self-confidence, career progression or salary [33, 36, 46, 47]. However, people with IA valued working to maintain a normal life [31, 39, 43–45, 50], financial security [39, 44], self-esteem, identity [43] and social networks [43]. Disability pensions were seen as a “last resort” [39]. At least 34% percent experienced difficulties at work, increased absenteeism in 11% and premature retirement in 8–18% [33, 38] with 25% worrying about job losses [44]. The need for modified footwear impacts on job roles in those with PsA and foot disease (e.g. unable to wear dress shoes or safety boots) [36]. Factors improving work retention included physical ability [51], travel arrangements (parking, working from home) [50], flexibility of hours and conditions [45, 50] and modifications in the workplace [45, 50]. Participants wanted more information about their work-related rights [39, 50].

5) Exercise and leisure related needs

Five OA studies (Table 2) [22, 24, 25, 28] identified barriers to exercise and leisure participation. Disease progression meant people needed to modify or swap exercise/leisure activities [24]. People worried about their inability to exercise [25], especially pain, inability to continue activities previously enjoyed [28, 32], missing out on activities with others [22] and needing to engage in more sedentary activities [24].

Seven studies investigated needs and attitudes of people with IA to exercise and leisure activities (Table 3) [34–37, 40, 47, 49]. Participants felt empowered by information about type and necessity of physical exercise [34]. In RA, physical, psychological, functional and social benefits from exercise were identified [35, 40]; exercise was identified as a critical part of self-management [49]. However, RA limited mobility and caused pain, restricting participation in specific exercises/sports [37, 40], with some activities wholly inaccessible [35]. Some participants found RA symptoms to be a motivator to be active [49]. Social, family and leisure activities were affected, limiting sports, fitness, hobbies and spontaneity [47]. Participants preferred options where physical activity served an additional purpose e.g. social contact, transportation, dog walking [49]. Participants identified a lack of RA-specific exercise programs, with exercise programs and instructors failing to consider limitations imposed by arthritis [40] or hesitation about joining general exercise classes due to being unable to keep up or lack of understanding [49]. Patients with PsA and foot disease struggled with walking, particularly on uneven ground [36].

6) Transport needs and environmental modification

Four OA studies (Table 2) [21, 24, 25, 28] investigated needs related to transport. More than 20% of people experienced difficulties in one study [21]. Services to increase accessibility to public transport may improve people’s ability to socialise [25]. In people with hand OA, supports were needed to facilitate opening doors, holding the steering wheel and using bicycle hand brakes [24], with use of public transportation limited by their inability to hold straps/poles [24]. Travel required significant planning, including in getting in and out of cars, finding seated toilets in Taiwan and needing plan analgesia around going out [28].

Five IA studies (Table 3) [40, 41, 44, 47, 51] identified environmental limitations to moving about outside the home. These included lack of appropriate transport [44] and unsafe environment related to fear of falling related to environmental factors (uneven pavement, lack of ramps and lighting) [40, 41, 44]. Driving could be difficult [47, 51]. Some participants worried about falling victim of crime due to frailty [44].

## Discussion

This review demonstrates the pervasive impact of arthritis on peoples’ lives, independent of aetiology. We have identified six key domains in which arthritis impacts life: daily living, social participation, financial security, occupation, exercise/leisure and transportation. All areas of need identified were common to OA and IA, illustrating that need appears to primarily be linked to symptoms common across musculoskeletal conditions, rather than aetiology or pathogenesis.

Non-healthcare needs related maintain daily functioning were identified by people with both OA and IA. Deficits lead to a sense of “failure”, particularly being unable to do household chores or care for children [22]. The pervasiveness of this theme highlights the importance of supporting functional ability in both ADLs and the workplace. People with arthritis appear to need robust social support systems to assist them with ADLs [22], and targeted assistive devices (e.g. for cooking) to enable participants to complete tasks and feel “normal” [23, 24, 40].

Needs related to social functioning were similar in people with IA and OA. Loneliness, withdrawal [24, 25, 43, 44], and lack of understanding from family, friends and communities were troublesome [23, 43], but partly negated by peer support groups [23]. People with both IA and OA wanted means to reduce social isolation. In the wider literature, social connectedness and “diffuse social relationships” have been identified as crucial to psychological wellbeing [54]. An individual’s health can be related to the strength of their social relationships, with participants in this review with “strong” social supports more likely to perceive good health [26]. Interestingly, in one study people with IA tended to be more interested (44%) in peer support groups than those with OA (20%) [31]. Further data are required regarding optimal delivery of peer support groups, particularly as self-management education groups are limited in effectiveness for most clinical outcomes [55].

The need for work retention was critical to people with both OA and IA, for financial security and social connectedness [43]. Arthritis is associated with reduced work productivity, early retirement and reduced wealth [6]. Flexibility and environmental adaptations in the workplace facilitated work retention in people with IA and OA [24, 25]. Maintaining employment is a key issue in people with RA, facilitated by both environmental adaptations and flexible work hours [56]. Use of modified schedules when required was associated with lower workplace activity limitation, fewer job disruptions and productivity losses [56]. Furthermore, loss of work or retirement exacerbated feelings of social isolation, highlighting the importance of employment in social connectedness and self-worth [43, 45]. Programs targeted to improve work retention in people with arthritis can reduce anxiety, improve mood and life satisfaction [57]. Given the importance of employment in financial wellbeing and social connectedness, further work is needed to identify contributors to work retention in people with arthritis and to support and educate employers and practitioners in providing these.

Both OA and IA had similar detrimental effects on physical and leisure activity participation. When activities couldn’t be modified/replaced, participants were excluded from activities with family and friends [22, 40]. Arthritis patients need assistance from healthcare providers with arthritis-specific exercise programs [58], as well as information about benefits and safety of exercise [34]. People with OA frequently report mobility and pain as barriers to participation in exercise [59], despite high quality evidence for its therapeutic benefits in OA and IA [60]. Access to practitioners with skills and knowledge in behaviour change, pain science and appropriate exercise programs/facilities is important for people with arthritis.

Transportation needs for those with IA and OA had broader impacts on other areas of need. Difficulties with transportation exacerbate dependence on others [40, 41], unemployment [50] and social isolation [32]. Transportation is intimately linked with freedom and independence in older adults, and has a pervasive impact on life [61]. Further research is required to understand factors limiting transportation and improve uptake in people with arthritis.

We have identified some contrasts between the experiences of people with OA and IA. Those with OA identified a lack of acknowledgement and community support [23, 43]. They felt OA and the resultant disability were underestimated with limited media coverage of, and research into, OA [23]. While participants with IA felt that sometimes their symptoms were underestimated [47], they did not report trivialisation of the disease itself. In the wider literature people with RA felt inadequate support and information were available, particularly in specific situations like pregnancy [62]. Public health campaigns could assist with educating the wider community about arthritis and its impact.

This study has limitations. Firstly, although these data highlight the impact of OA and IA, existing literature focusses on problems related to arthritis rather than evaluation of actual needs. Thus, due to a paucity of data, we have not directly questioned the “needs” of people with OA and IA. It remains unknown which services exist and meet current needs, and which are insufficient, an important gap in the literature. This is a focus of the World Health Organisation’s Integrated Care for Older people approach. However, in line with the Gothenburg model of person-centred care, a key step in providers being able to deliver effective, person-centred care is to understand the experience of the person [63]. Accordingly, we believe summarising the literature regarding these issues is an important step towards addressing non-healthcare needs of people with arthritis, and enabling the delivery of effective person-centred care. Given the limited data, it is difficult to comment on whether non-healthcare needs differ according to country or social setting. Furthermore, as participants included mainly post-menopausal females, generalizability may be limited to other groups (particularly men, younger people, and those in low- and middle-income settings). Studies included modest sample sizes. Heterogeneity of data collected means different areas were investigated in each study; this provides limited triangulation and/or validation of any single conclusion. Overall studies were at moderate risk of bias, with higher risk of bias in data collection and recruitment. Finally, we did not perform inter-rater reliability for study selection.

This review has numerous strengths. A comprehensive scoping literature search was performed across four different databases. Many qualitative studies were included to enable deeper exploration of participants’ non-healthcare needs and perspectives. This search captured data from multiple levels of care, including community-based populations, as well as a range of different disease stages. OA studies involved a range of joints.

## Conclusions

Arthritis has a pervasive impact on different areas of life, regardless of disease aetiology. To patients, the similarities in functional impact far outweigh the differences in the disease pathogenesis. Whilst people with arthritis are acutely aware of their inability to perform tasks and perceived “failures”, little work has been performed to identify the patients’ perspective of non-healthcare needs to facilitate targeted service provision and provide holistic care. Future research is required to assess this, across a broader population and joint involvement, to identify whether there may be joint-specific non-healthcare needs. Improved characterization of the patients’ perceived non-healthcare needs is necessary to provide relevant support and services for people with arthritis.

## Supplementary Information

**Additional file 1.**

## Data Availability

All data generated or analysed during the current study are included in this published article and its supplementary files.

## ABBREVATIONS

ADLs: Activities of daily living
IA: Inflammatory arthritis
OA: Osteoarthritis
PsA: Psoriatic arthritis
RA: Rheumatoid arthritis
SLE: Systemic lupus erythematosus
