# Supplementary material for: Person-centred care in osteoarthritis and inflammatory arthritis: a scoping review of people’s needs outside of healthcare
Source: BMC Musculoskelet Disord. 2021 Apr 9;22:341. doi: 10.1186/s12891-021-04190-z (PMC8035722; doi:10.1186/s12891-021-04190-z)
Supplement: Supplementary file 1 — Additional file 1. [file 12891_2021_4190_MOESM1_ESM.docx]

Supplementary Electronic Material

**Supplementary Figure S1: Medline Search Strategy**

MEDLINE Search strategy for Patient perceived other service needs related to inflammatory arthritis

| 1. (consumer* or patient* or client* or customer* or service user*).tw. |  |
| --- | --- |
| 2. patients/ or inpatients/ or outpatients/ |  |
| 3. 1 or 2 |  |
| 4. ((work* or employ* or occupation* or job* or industr*) adj4 (participat* or retain* or retention or capacity or capability or function* or discriminat* or prejudic* or find* or keep* or gain* or obtain* or secur* or access* or opportunit* or return* or safe* or well* or hygiene or evaluat* or assess* or rehab*)).tw. |  |
| 5. (unemploy* or jobless*).tw. |  |
| 6. Occupational Health/ |  |
| 7. employment/ or employment, supported/ or return to work/ or unemployment/ or workplace/ |  |
| 8. exp Disability Evaluation/ |  |
| 9. (transport* or travel*).tw. |  |
| 10. exp Transportation/ |  |
| 11. travel/ or air travel/ |  |
| 12. posture/ and workplace/ |  |
| 13. (ergonomic* or posture*).tw. |  |
| 14. (architect* or space* or spacial* or facilit* or residen* or build* or home* or house* or apartment* or dwelling* or flat* or unit* or ramp* or park* or access* or barrier* or design* or product* or object*).tw. |  |
| 15. architectural accessibility/ or "elevators and escalators"/ or "floors and floorcoverings"/ or "interior design and furnishings"/ or parking facilities/ |  |
| 16. Environment Design/ |  |
| 17. ((self or independent or home) adj4 (care* or caring or life or lives or living or manag* or medica*)).tw. |  |
| 18. self care/ or self administration/ or self medication/ |  |
| 19. (activities of daily living or adl*).tw. |  |
| 20. exp "Activities of Daily Living"/ |  |
| 21. ((assist* or self help) adj3 (device* or technolog*)).tw. |  |
| 22. exp Self-Help Devices/ |  |
| 23. ((walk* or ambulat* or mobil*) adj3 (difficult* or limit* or impair*)).tw. |  |
| 24. mobility limitation/ |  |
| 25. ((social* or communit* or famil* or friend* or self help or peer* or colleague* or collegial* or therapeutic) adj4 (life* or live* or participat* or adjust* or accept* or distan* or discriminat* or isolat* or relation* or welfare* or integrat* or dispute* or alien* or support* or assist* or group* or club* or organi#ation* or network* or strain* or stress*)).tw. |  |
| 26. prejudice/ or social adjustment/ or social discrimination/ or social distance/ or social marginalization/ |  |
| 27. Community Integration/ or exp Interpersonal Relations/ or Self-Help Groups/ or peer group/ or Social Welfare/ |  |
| 28. social capital/ or exp social environment/ or exp social isolation/ or socialization/ |  |
| 29. (care giver* or caregiver* or respite or (home* adj3 (nurs* or assist* or help*)) or child care or childcare).tw. |  |
| 30. Caregivers/ or exp Home Nursing/ or child care/ or infant care/ |  |
| 31. ((financ* or monetary or economic*) adj4 (supp* or assist* or subsid*)).tw. |  |
| 32. financial support/ |  |
| 33. ((civil* or consumer* or patient*) adj4 (right* or advoca* or justice)).tw. |  |
| 34. (legislat* or jurisprudence or law* or legal*).tw. |  |
| 35. civil rights/ or consumer advocacy/ or patient rights/ or jurisprudence/ or patient advocacy/ or social justice/ or legislation as topic/ |  |
| 36. or/4-35 |  |
| 37. (utili* or need* or seek* or retriev* or provid* or provision or source* or aid* or promot* or access* or demand* or insufficien* or deficit* or gap* or barrier* or enabler* or facilitat* or deliver* or implement* or manag* or coordinat*).tw. |  |
| 38. Needs Assessment/ |  |
| 39. 37 or 38 |  |
| 40. ((consumer* or patient* or client* or customer* or service user*) adj4 (need* or want* or like* or interest* or prefer* or satisf* or perspective* or experience* or attitude* or belief* or practice* or concern* or support* or participat* or advoca* or center* or centr* or orient* or focus* or empower* or expect* or opinion* or view* or perceive* or perception* or tailor* or bespoke or involv* or priorit* or control*)).tw. |  |
| 41. patient preference/ or patient satisfaction/ or Health Knowledge, Attitudes, Practice/ |  |
| 42. 40 or 41 |  |
| 43. 3 and 36 and 39 and 42 |  |
| 44. 41 and 43 |  |
| 45. exp rheumatoid arthritis/ |  |
| 46. (felty* adj2 syndrome).tw. |  |
| 47. (caplan* adj2 syndrome).tw. |  |
| 48. (sjogren* adj2 syndrome).tw. |  |
| 49. (sicca adj2 syndrome).tw. |  |
| 50. (ankylos* or spondyl*).tw. |  |
| 51. (psoria* adj2 arthr*).tw. |  |
| 52. reactive arthritis.tw. |  |
| 53. (reiter* adj (disease or syndrome)).tw. |  |
| 54. enthesi*.tw. |  |
| 55. inflammatory arthritis.tw. |  |
| 56. ((sexua* or chlamydia or yersinia or postyersinia or postdysenteric or salmnella or shigella or b27 or postinfectious or post infectious) adj5 arthr*).tw. |  |
| 57. (rheumat* adj3 (arthr* or diseas* or condition* or nodule*)).tw. |  |
| 58. (bechtere* disease* or marie-struempell disease* or rheumatoid spondylitis or spondylarthritis ankylopoietica or ankylo* spondyl* or Spin* Ankylosis or Vertebral Ankylosis).tw. |  |
| 59. sacroiliitis.tw. |  |
| 60. dactylit*.tw. |  |
| 61. Uveitis.tw. |  |
| 62. Iritis.tw. |  |
| 63. 45 or 46 or 47 or 48 or 49 or 50 or 51 or 52 or 53 or 54 or 55 or 56 or 57 or 58 or 59 or 60 or 61 or 62 |  |
| 64. 44 and 63 |  |

MEDLINE Search strategy for patient perceived Other Service Needs And OA MEDLINE

| 1. (consumer* or patient* or client* or customer* or service user*).tw. |  |
| --- | --- |
| 2. patients/ or inpatients/ or outpatients/ |  |
| 3. 1 or 2 |  |
| 4. ((work* or employ* or occupation* or job* or industr*) adj4 (participat* or retain* or retention or capacity or capability or function* or discriminat* or prejudic* or find* or keep* or gain* or obtain* or secur* or access* or opportunit* or return* or safe* or well* or hygiene or evaluat* or assess* or rehab*)).tw. |  |
| 5. (unemploy* or jobless*).tw. |  |
| 6. Occupational Health/ |  |
| 7. employment/ or employment, supported/ or return to work/ or unemployment/ or workplace/ |  |
| 8. exp Disability Evaluation/ |  |
| 9. (transport* or travel*).tw. |  |
| 10. exp Transportation/ |  |
| 11. travel/ or air travel/ |  |
| 12. posture/ and workplace/ |  |
| 13. (ergonomic* or posture*).tw. |  |
| 14. (architect* or space* or spacial* or facilit* or residen* or build* or home* or house* or apartment* or dwelling* or flat* or unit* or ramp* or park* or access* or barrier* or design* or product* or object*).tw. |  |
| 15. architectural accessibility/ or "elevators and escalators"/ or "floors and floorcoverings"/ or "interior design and furnishings"/ or parking facilities/ |  |
| 16. Environment Design/ |  |
| 17. ((self or independent or home) adj4 (care* or caring or life or lives or living or manag* or medica*)).tw. |  |
| 18. self care/ or self administration/ or self medication/ |  |
| 19. (activities of daily living or adl*).tw. |  |
| 20. exp "Activities of Daily Living"/ |  |
| 21. ((assist* or self help) adj3 (device* or technolog*)).tw. |  |
| 22. exp Self-Help Devices/ |  |
| 23. ((walk* or ambulat* or mobil*) adj3 (difficult* or limit* or impair*)).tw. |  |
| 24. mobility limitation/ |  |
| 25. ((social* or communit* or famil* or friend* or self help or peer* or colleague* or collegial* or therapeutic) adj4 (life* or live* or participat* or adjust* or accept* or distan* or discriminat* or isolat* or relation* or welfare* or integrat* or dispute* or alien* or support* or assist* or group* or club* or organi#ation* or network* or strain* or stress*)).tw. |  |
| 26. prejudice/ or social adjustment/ or social discrimination/ or social distance/ or social marginalization/ |  |
| 27. Community Integration/ or exp Interpersonal Relations/ or Self-Help Groups/ or peer group/ or Social Welfare/ |  |
| 28. social capital/ or exp social environment/ or exp social isolation/ or socialization/ |  |
| 29. (care giver* or caregiver* or respite or (home* adj3 (nurs* or assist* or help*)) or child care or childcare).tw. |  |
| 30. Caregivers/ or exp Home Nursing/ or child care/ or infant care/ |  |
| 31. ((financ* or monetary or economic*) adj4 (supp* or assist* or subsid*)).tw. |  |
| 32. financial support/ |  |
| 33. ((civil* or consumer* or patient*) adj4 (right* or advoca* or justice)).tw. |  |
| 34. (legislat* or jurisprudence or law* or legal*).tw. |  |
| 35. civil rights/ or consumer advocacy/ or patient rights/ or jurisprudence/ or patient advocacy/ or social justice/ or legislation as topic/ |  |
| 36. or/4-35 |  |
| 37. (utili* or need* or seek* or retriev* or provid* or provision or source* or aid* or promot* or access* or demand* or insufficien* or deficit* or gap* or barrier* or enabler* or facilitat* or deliver* or implement* or manag* or coordinat*).tw. |  |
| 38. Needs Assessment/ |  |
| 39. 37 or 38 |  |
| 40. ((consumer* or patient* or client* or customer* or service user*) adj4 (need* or want* or like* or interest* or prefer* or satisf* or perspective* or experience* or attitude* or belief* or practice* or concern* or support* or participat* or advoca* or center* or centr* or orient* or focus* or empower* or expect* or opinion* or view* or perceive* or perception* or tailor* or bespoke or involv* or priorit* or control*)).tw. |  |
| 41. patient preference/ or patient satisfaction/ or Health Knowledge, Attitudes, Practice/ |  |
| 42. 40 or 41 |  |
| 43. 3 and 36 and 39 and 42 |  |
| 44. 41 and 43 |  |
| 45. exp osteoarthritis/ |  |
| 46. (degen* adj4 arth*).tw. |  |
| 47. osteoarth*.tw. |  |
| 48. coxarth*.tw. |  |
| 49. gonarth*.tw. |  |
| 50. 45 or 46 or 47 or 48 or 49 |  |
| 51. 44 and 50 |  |

**Figure S2a: EMBASE Search Strategy: non-healthcare needs and inflammatory arthritis**

| 1. patient/ or hospital patient/ or outpatient/ |  |  |
| --- | --- | --- |
| 2. (consumer* or patient* or client* or customer* or service user*).tw. | | |
| 3. 1 or 2 | | |
| 18. environmental planning/ | | |
| 19. ((self or independent or home) adj4 (care* or caring or life or lives or living or manag* or medica*)).tw. | | |
| 20. drug self administration/ | | |
| 21. self care/ or self care agency/ or self help/ or self medication/ | | |
| 22. (activities of daily living or adl*).tw. | | |
| 23. daily life activity/ | | |
| 24. ((assist* or self help) adj3 (device* or technolog*)).tw. | | |
| 25. self help device/ | | |
| 26. ((walk* or ambulat* or mobil*) adj3 (difficult* or limit* or impair*)).tw. | | |
| 27. walking difficulty/ | | |
| 28. ((social* or communit* or famil* or friend* or self help or peer* or colleague* or collegial* or therapeutic) adj4 (life* or live* or participat* or adjust* or accept* or distan* or discriminat* or isolat* or relation* or welfare* or integrat* or dispute* or alien* or support* or assist* or group* or club* or organi#ation* or network* or strain* or stress*)).tw. | | |
| 29. prejudice/ | | |
| 30. social adaptation/ | | |
| 31. social discrimination/ | | |
| 32. social distance/ | | |
| 33. community integration/ | | |
| 34. human relation/ | | |
| 35. self help/ | | |
| 36. peer group/ | | |
| 37. social welfare/ | | |
| 38. social environment/ or psychosocial environment/ or social capital/ | | |
| 39. social exclusion/ | | |
| 40. socialization/ | | |
| 41. (care giver* or caregiver* or respite or (home* adj3 (nurs* or assist* or help*)) or child care or childcare).tw. | | |
| 42. caregiver/ | | |
| 43. home care/ | | |
| 44. child care/ | | |
| 45. ((financ* or monetary or economic*) adj4 (supp* or assist* or subsid*)).tw. | | |
| 46. financial management/ | | |
| 47. ((civil* or consumer* or patient*) adj4 (right* or advoca* or justice)).tw. | | |
| 48. (legislat* or jurisprudence or law* or legal*).tw. | | |
| 49. civil rights/ | | |
| 50. patient right/ | | |
| 51. consumer advocacy/ | | |
| 52. jurisprudence/ | | |
| 53. patient advocacy/ | | |
| 54. social justice/ | | |
| 55. law/ | | |
| 56. or/4-55 | | |
| 57. (utili* or need* or seek* or retriev* or provid* or provision or source* or aid* or promot* or access* or demand* or insufficien* or deficit* or gap* or barrier* or enabler* or facilitat* or deliver* or implement* or manag* or coordinat*).tw. | | |
| 58. needs assessment/ | | |
| 59. health care delivery/ | | |
| 60. 57 or 58 or 59 | | |
| 61. ((consumer* or patient* or client* or customer* or service user*) adj4 (need* or want* or like* or interest* or prefer* or satisf* or perspective* or experience* or attitude* or belief* or practice* or concern* or support* or participat* or advoca* or center* or centr* or orient* or focus* or empower* or expect* or opinion* or view* or perceive* or perception* or tailor* or bespoke or involv* or priorit* or control*)).tw. | | |
| 62. patient care/ | | |
| 63. patient attitude/ or patient preference/ or patient satisfaction/ | | |
| 64. attitude to health/ | | |
| 65. 62 or 63 or 64 | | |
| 66. 61 or 65 | | |
| 67. 3 and 56 and 60 and 66 | | |
| 68. 65 and 67 | | |
| 69. exp rheumatoid arthritis/ | | |
| 70. (felty* adj2 syndrome).tw. | | |
| 71. (caplan* adj2 syndrome).tw. | | |
| 72. (sjogren* adj2 syndrome).tw. | | |
| 73. (sicca adj2 syndrome).tw. | | |
| 74. spondyloarthropathy/ | | |
| 75. (ankylos* or spondyl*).tw. | | |
| 76. psoriatic arthritis/ | | |
| 77. (psoria* adj2 arthr*).tw. | | |
| 78. reactive arthritis.tw. | | |
| 79. (reiter* adj (disease or syndrome)).tw. | | |
| 80. enthesi*.tw. | | |
| 81. inflammatory arthritis.tw. | | |
| 82. ((sexua* or chlamydia or yersinia or postyersinia or postdysenteric or salmnella or shigella or b27 or postinfectious or post infectious) adj5 arthr*).tw. | | |
| 83. (rheumat* adj3 (arthr* or diseas* or condition* or nodule*)).tw. | | |
| 84. ankylosing spondylitis/ | | |
| 85. (bechtere* disease* or marie-struempell disease* or rheumatoid spondylitis or spondylarthritis ankylopoietica or ankylo* spondyl* or Spin* Ankylosis or Vertebral Ankylosis).tw. | | |
| 86. sacroiliitis/ | | |
| 87. sacroiliitis.tw. | | |
| 88. HLA B27 antigen/ | | |
| 89. dactylit*.tw. | | |
| 90. iritis/ | | |
| 91. Iritis.tw. | | |
| 92. uveitis/ | | |
| 93. Uveitis.tw. | | |
| 94. 69 or 70 or 71 or 72 or 73 or 74 or 75 or 76 or 77 or 78 or 79 or 80 or 81 or 82 or 83 or 84 or 85 or 86 or 87 or 88 or 89 or 90 or 91 or 92 or 93 | | |
| 95. 68 and 94 | | |

**Figure S2b: EMBASE search strategy: Non-healthcare needs and Osteoarthritis**

| 1. patient/ or hospital patient/ or outpatient/ |
| --- |
| 2. (consumer* or patient* or client* or customer* or service user*).tw. |
| 3. 1 or 2 |
| 4. ((work* or employ* or occupation* or job* or industr*) adj4 (participat* or retain* or retention or capacity or capability or function* or discriminat* or prejudic* or find* or keep* or gain* or obtain* or secur* or access* or opportunit* or return* or safe* or well* or hygiene or evaluat* or assess* or rehab*)).tw. |
| 5. (unemploy* or jobless*).tw. |
| 6. occupational health/ or employability/ or job accommodation/ or "quality of working life"/ or vocational rehabilitation/ or work capacity/ or work resumption/ |
| 7. employment/ or employment status/ |
| 8. workplace/ |
| 9. disability/ or physical disability/ or walking difficulty/ or work disability/ |
| 10. 4 or 5 or 6 or 7 or 8 or 9 |
| 11. (transport* or travel*).tw. |
| 12. "traffic and transport"/ |
| 13. posture/ and workplace/ |
| 14. (ergonomic* or posture*).tw. |
| 15. (architect* or space* or spacial* or facilit* or residen* or build* or home* or house* or apartment* or dwelling* or flat* or unit* or ramp* or park* or access* or barrier* or design* or product* or object*).tw. |
| 16. "construction work and architectural phenomena"/ or architectural barrier/ |
| 17. building/ |
| 18. environmental planning/ |
| 19. ((self or independent or home) adj4 (care* or caring or life or lives or living or manag* or medica*)).tw. |
| 20. drug self administration/ |
| 21. self care/ or self care agency/ or self help/ or self medication/ |
| 22. (activities of daily living or adl*).tw. |
| 23. daily life activity/ |
| 24. ((assist* or self help) adj3 (device* or technolog*)).tw. |
| 25. self help device/ |
| 26. ((walk* or ambulat* or mobil*) adj3 (difficult* or limit* or impair*)).tw. |
| 27. walking difficulty/ |
| 28. ((social* or communit* or famil* or friend* or self help or peer* or colleague* or collegial* or therapeutic) adj4 (life* or live* or participat* or adjust* or accept* or distan* or discriminat* or isolat* or relation* or welfare* or integrat* or dispute* or alien* or support* or assist* or group* or club* or organi#ation* or network* or strain* or stress*)).tw. |
| 29. prejudice/ |
| 30. social adaptation/ |
| 31. social discrimination/ |
| 32. social distance/ |
| 33. community integration/ |
| 34. human relation/ |
| 35. self help/ |
| 36. peer group/ |
| 37. social welfare/ |
| 38. social environment/ or psychosocial environment/ or social capital/ |
| 39. social exclusion/ |
| 40. socialization/ |
| 41. (care giver* or caregiver* or respite or (home* adj3 (nurs* or assist* or help*)) or child care or childcare).tw. |
| 42. caregiver/ |
| 43. home care/ |
| 44. child care/ |
| 45. ((financ* or monetary or economic*) adj4 (supp* or assist* or subsid*)).tw. |
| 46. financial management/ |
| 47. ((civil* or consumer* or patient*) adj4 (right* or advoca* or justice)).tw. |
| 48. (legislat* or jurisprudence or law* or legal*).tw. |
| 49. civil rights/ |
| 50. patient right/ |
| 51. consumer advocacy/ |
| 52. jurisprudence/ |
| 53. patient advocacy/ |
| 54. social justice/ |
| 55. law/ |
| 56. or/4-55 |
| 57. (utili* or need* or seek* or retriev* or provid* or provision or source* or aid* or promot* or access* or demand* or insufficien* or deficit* or gap* or barrier* or enabler* or facilitat* or deliver* or implement* or manag* or coordinat*).tw. |
| 58. needs assessment/ |
| 59. health care delivery/ |
| 60. 57 or 58 or 59 |
| 61. ((consumer* or patient* or client* or customer* or service user*) adj4 (need* or want* or like* or interest* or prefer* or satisf* or perspective* or experience* or attitude* or belief* or practice* or concern* or support* or participat* or advoca* or center* or centr* or orient* or focus* or empower* or expect* or opinion* or view* or perceive* or perception* or tailor* or bespoke or involv* or priorit* or control*)).tw. |
| 62. patient care/ |
| 63. patient attitude/ or patient preference/ or patient satisfaction/ |
| 64. attitude to health/ |
| 65. 62 or 63 or 64 |
| 66. 61 or 65 |
| 67. 3 and 56 and 60 and 66 |
| 68. 65 and 67 |
| 69. exp osteoarthritis/ |
| 70. (degen* adj4 arth*).tw. |
| 71. osteoarth*.tw. |
| 72. coxarth*.tw. |
| 73. gonarth*.tw. |
| 74. 69 or 70 or 71 or 72 or 73 |
| 75. 68 and 74 |

**Figure S3a: PsychINFO search strategy: Non-healthcare needs and Inflammatory Arthritis**

| 1. patients/ or hospitalized patients/ or outpatients/ |
| --- |
| 2. (consumer* or patient* or client* or customer*).tw. |
| 3. 1 or 2 |
| 4. ((work* or employ* or occupation* or job* or industr*) adj4 (participat* or retain* or retention or capacity or capability or function* or discriminat* or prejudic* or find* or keep* or gain* or obtain* or secur* or access* or opportunit* or return* or safe* or well* or hygiene or evaluat* or assess* or rehab*)).tw. |
| 5. (unemploy* or jobless*).tw. |
| 6. occupational health/ |
| 7. employment status/ or self employment/ or unemployment/ or employability/ or reemployment/ |
| 8. disability evaluation/ |
| 9. exp transportation/ |
| 10. (ergonomic* or posture*).tw. |
| 11. (architect* or space* or spacial* or facilit* or residen* or build* or home* or house* or apartment* or dwelling* or flat* or unit* or ramp* or park* or access* or barrier* or design* or product* or object*).tw. |
| 12. exp "accommodation (disabilities)"/ |
| 13. built environment/ |
| 14. ((self or independent or home) adj4 (care* or caring or life or lives or living or manag* or medica*)).tw. |
| 15. self care skills/ or "activities of daily living"/ or adaptive behavior/ |
| 16. drug self administration/ |
| 17. self medication/ |
| 18. (activities of daily living or adl*).tw. |
| 19. mobility aids/ or assistive technology/ |
| 20. ((assist* or self help) adj3 (device* or technolog*)).tw. |
| 21. ((walk* or ambulat* or mobil*) adj3 (difficult* or limit* or impair*)).tw. |
| 22. ((social* or communit* or famil* or friend* or self help or peer* or colleague* or collegial* or therapeutic) adj4 (life* or live* or participat* or adjust* or accept* or distan* or discriminat* or isolat* or relation* or welfare* or integrat* or dispute* or alien* or support* or assist* or group* or club* or organi#ation* or network* or strain* or stress*)).tw. |
| 23. prejudice/ or disability discrimination/ or employment discrimination/ |
| 24. social adjustment/ |
| 25. social integration/ |
| 26. interpersonal relationships/ or family relations/ or marital relations/ or interpersonal interaction/ or psychological distance/ or relationship quality/ or relationship satisfaction/ |
| 27. (care giver* or caregiver* or respite or (home* adj3 (nurs* or assist* or help*)) or child care or childcare).tw. |
| 28. home care/ |
| 29. child care/ or caregivers/ |
| 30. ((financ* or monetary or economic*) adj4 (supp* or assist* or subsid*)).tw. |
| 31. financial services/ |
| 32. ((civil* or consumer* or patient*) adj4 (right* or advoca* or justice)).tw. |
| 33. (legislat* or jurisprudence or law* or legal*).tw. |
| 34. "law (government)"/ |
| 35. civil rights/ or client rights/ or advocacy/ or civil law/ or disability laws/ or social equality/ or social justice/ |
| 36. or/4-35 |
| 37. (utili* or need* or seek* or retriev* or provid* or provision or source* or aid* or promot* or access* or demand* or insufficien* or deficit* or gap* or barrier* or enabler* or facilitat* or deliver* or implement* or manag* or coordinat*).tw. |
| 38. needs assessment/ or special needs/ |
| 39. 37 or 38 |
| 40. ((consumer* or patient* or client* or customer* or service user*) adj4 (need* or want* or like* or interest* or prefer* or satisf* or perspective* or experience* or attitude* or belief* or practice* or concern* or support* or participat* or advoca* or center* or centr* or orient* or focus* or empower* or expect* or opinion* or view* or perceive* or perception* or tailor* or bespoke or involv* or priorit* or control*)).tw. |
| 41. preferences/ |
| 42. client satisfaction/ |
| 43. health knowledge/ or health attitudes/ |
| 44. 41 or 42 or 43 |
| 45. 40 or 44 |
| 46. 3 and 36 and 38 and 45 |
| 47. exp rheumatoid arthritis/ |
| 48. (felty* adj2 syndrome).tw. |
| 49. (caplan* adj2 syndrome).tw. |
| 50. (sjogren* adj2 syndrome).tw. |
| 51. (sicca adj2 syndrome).tw. |
| 52. (ankylos* or spondyl*).tw. |
| 53. (psoria* adj2 arthr*).tw. |
| 54. reactive arthritis.tw. |
| 55. (reiter* adj (disease or syndrome)).tw. |
| 56. enthesi*.tw. |
| 57. inflammatory arthritis.tw. |
| 58. ((sexua* or chlamydia or yersinia or postyersinia or postdysenteric or salmnella or shigella or b27 or postinfectious or post infectious) adj5 arthr*).tw. |
| 59. (rheumat* adj3 (arthr* or diseas* or condition* or nodule*)).tw. |
| 60. (bechtere* disease* or marie-struempell disease* or rheumatoid spondylitis or spondylarthritis ankylopoietica or ankylo* spondyl* or Spin* Ankylosis or Vertebral Ankylosis).tw. |
| 61. sacroiliitis.tw. |
| 62. dactylit*.tw. |
| 63. Uveitis.tw. |
| 64. Iritis.tw. |
| 65. 47 or 48 or 49 or 50 or 51 or 52 or 53 or 54 or 55 or 56 or 57 or 58 or 59 or 60 or 61 or 62 or 63 or 64 |
| 66. 46 and 65 |

**Figure S3b: PsychINFO Search Strategy - Non-healthcare needs and osteoarthritis**

| 1. patients/ or hospitalized patients/ or outpatients/ |  |
| --- | --- |
| 2. (consumer* or patient* or client* or customer*).tw. |  |
| 3. 1 or 2 |  |
| 4. ((work* or employ* or occupation* or job* or industr*) adj4 (participat* or retain* or retention or capacity or capability or function* or discriminat* or prejudic* or find* or keep* or gain* or obtain* or secur* or access* or opportunit* or return* or safe* or well* or hygiene or evaluat* or assess* or rehab*)).tw. |  |
| 5. (unemploy* or jobless*).tw. |  |
| 7. employment status/ or self employment/ or unemployment/ or employability/ or reemployment/ |  |
| 8. disability evaluation/ |  |
| 9. exp transportation/ |  |
| 10. (ergonomic* or posture*).tw. |  |
| 11. (architect* or space* or spacial* or facilit* or residen* or build* or home* or house* or apartment* or dwelling* or flat* or unit* or ramp* or park* or access* or barrier* or design* or product* or object*).tw. |  |
| 12. exp "accommodation (disabilities)"/ |  |
| 13. built environment/ |  |
| 14. ((self or independent or home) adj4 (care* or caring or life or lives or living or manag* or medica*)).tw. |  |
| 15. self care skills/ or "activities of daily living"/ or adaptive behavior/ |  |
| 16. drug self administration/ |  |
| 17. self medication/ |  |
| 18. (activities of daily living or adl*).tw. |  |
| 19. mobility aids/ or assistive technology/ |  |
| 20. ((assist* or self help) adj3 (device* or technolog*)).tw. |  |
| 21. ((walk* or ambulat* or mobil*) adj3 (difficult* or limit* or impair*)).tw. |  |
| 22. ((social* or communit* or famil* or friend* or self help or peer* or colleague* or collegial* or therapeutic) adj4 (life* or live* or participat* or adjust* or accept* or distan* or discriminat* or isolat* or relation* or welfare* or integrat* or dispute* or alien* or support* or assist* or group* or club* or organi#ation* or network* or strain* or stress*)).tw. |  |
| 23. prejudice/ or disability discrimination/ or employment discrimination/ |  |
| 24. social adjustment/ |  |
| 25. social integration/ |  |
| 26. interpersonal relationships/ or family relations/ or marital relations/ or interpersonal interaction/ or psychological distance/ or relationship quality/ or relationship satisfaction/ |  |
| 27. (care giver* or caregiver* or respite or (home* adj3 (nurs* or assist* or help*)) or child care or childcare).tw. |  |
| 28. home care/ |  |
| 29. child care/ or caregivers/ |  |
| 30. ((financ* or monetary or economic*) adj4 (supp* or assist* or subsid*)).tw. |  |
| 31. financial services/ |  |
| 32. ((civil* or consumer* or patient*) adj4 (right* or advoca* or justice)).tw. |  |
| 33. (legislat* or jurisprudence or law* or legal*).tw. |  |
| 34. "law (government)"/ |  |
| 35. civil rights/ or client rights/ or advocacy/ or civil law/ or disability laws/ or social equality/ or social justice/ |  |
| 36. or/4-35 |  |
| 37. (utili* or need* or seek* or retriev* or provid* or provision or source* or aid* or promot* or access* or demand* or insufficien* or deficit* or gap* or barrier* or enabler* or facilitat* or deliver* or implement* or manag* or coordinat*).tw. |  |
| 38. needs assessment/ or special needs/ |  |
| 39. 37 or 38 |  |
| 40. ((consumer* or patient* or client* or customer* or service user*) adj4 (need* or want* or like* or interest* or prefer* or satisf* or perspective* or experience* or attitude* or belief* or practice* or concern* or support* or participat* or advoca* or center* or centr* or orient* or focus* or empower* or expect* or opinion* or view* or perceive* or perception* or tailor* or bespoke or involv* or priorit* or control*)).tw. |  |
| 41. preferences/ |  |
| 42. client satisfaction/ |  |
| 43. health knowledge/ or health attitudes/ |  |
| 44. 41 or 42 or 43 |  |
| 45. 40 or 44 |  |
| 46. 3 and 36 and 38 and 45 |  |
| 47. osteoarth*.tw. |  |
| 48. (degen* adj4 arth*).tw. |  |
| 49. coxarth*.tw. |  |
| 50. gonarth*.tw. |  |
| 51. Arthritis/ |  |
| 52. 47 or 48 or 49 or 50 or 51 |  |
| 53. 46 and 52 |  |

**Figure S4a: CINAHL Search Strategy: non-healthcare needs and inflammatory arthritis**

**# Query**

S147 S123 AND S146

S146 S124 OR S125 OR S126 OR S127 OR S128 OR S129 OR S130 OR S131 OR S132 OR S133 OR S134 OR S135 OR S136 OR S137 OR S138 OR S139 OR S140 OR S141 OR S142 OR S143 OR S144 OR S145

S145 (MH "Reiter Disease")

S144 (MH "Arthritis, Psoriatic")

S143 (MH "Iritis")

S142 (MH "Uveitis")

S141 TI Iritis OR AB Iritis

S140 TI Uveitis OR AB Uveitis

S139 TI dactylit* OR AB dactylit*

S138 TI sacroiliitis OR AB sacroiliitis

S137 TI (bechtere* disease* or marie-struempell disease* or rheumatoid spondylitis or spondylarthritis ankylopoietica or ankylo* spondyl* or Spin* Ankylosis or Vertebral Ankylosis) OR AB (bechtere* disease* or marie-struempell disease* or rheumatoid spondylitis or spondylarthritis ankylopoietica or ankylo* spondyl* or Spin* Ankylosis or Vertebral Ankylosis)

S136 TI (rheumat* N3 (arthr* or diseas* or condition* or nodule*)) OR AB (rheumat* N3 (arthr* or diseas* or condition* or nodule*))

S135 TI ((sexua* or chlamydia or yersinia or postyersinia or postdysenteric or salmnella or shigella or b27 or postinfectious or post infectious) N5 arthr*) OR AB ((sexua* or chlamydia or yersinia or postyersinia or postdysenteric or salmnella or shigella or b27 or postinfectious or post infectious) N5 arthr*)

S134 TI (inflammatory N2 arthritis) OR AB (inflammatory N2 arthritis)

S133 TI enthesi* OR AB enthesi*

S132 TI (reiter* N1 (disease or syndrome)) OR AB (reiter* N1 (disease or syndrome))

S131 TI "reactive arthritis" OR AB "reactive arthritis"

S130 TI (psoria* N2 arthr*) OR AB (psoria* N2 arthr*)

S129 TI (ankylos* or spondyl*) OR AB (ankylos* or spondyl*)

S128 TI (sicca N2 syndrome) OR AB (sicca N2 syndrome)

S127 TI (sjogren* N2 syndrome) OR AB (sjogren* N2 syndrome)

S126 TI (caplan* N2 syndrome) OR AB (caplan* N2 syndrome)

S125 TI (felty* N2 syndrome) OR AB (felty* N2 syndrome)

S124 (MH "Arthritis, Rheumatoid+")

S123 S69 AND S114 AND S118 AND S122

S122 S119 OR S120 OR S121

S121 (MH "Patient Centered Care")

S120 (MH "Consumer Satisfaction") OR (MH "Patient Satisfaction")

S119 TI (consumer* or patient* or client* or customer* or service user*) N4 (need* or want* or like* or interest* or prefer* or satisf* or perspective* or experience* or attitude* or belief* or practice* or concern* or support* or participat* or advoca* or center* or centr* or orient* or focus* or empower* or expect* or opinion* or view* or perceive* or perception* or tailor* or bespoke or involv* or priorit* or control*) OR AB (consumer* or patient* or client* or customer* or service user*) N4 (need* or want* or like* or interest* or prefer* or satisf* or perspective* or experience* or attitude* or belief* or practice* or concern* or support* or participat* or advoca* or center* or centr* or orient* or focus* or empower* or expect* or opinion* or view* or perceive* or perception* or tailor* or bespoke or involv* or priorit* or control*)

S118 S115 OR S116

S116 (MH "Needs Assessment")

S115 TI (utili* or need* or seek* or retriev* or provid* or provision or source* or aid* or promot* or access* or demand* or insufficien* or deficit* or gap* or barrier* or enabler* or facilitat* or deliver* or implement* or manag* or coordinat*) OR AB (utili* or need* or seek* or retriev* or provid* or provision or source* or aid* or promot* or access* or demand* or insufficien* or deficit* or gap* or barrier* or enabler* or facilitat* or deliver* or implement* or manag* or coordinat*)

S114 S70 OR S71 OR S72 OR S73 OR S74 OR S75 OR S76 OR S77 OR S78 OR S79 OR S80 OR S81 OR S82 OR S83 OR S84 OR S85 OR S86 OR S87 OR S88 OR S89 OR S90 OR S91 OR S92 OR S93 OR S94 OR S95 OR S96 OR S97 OR S98 OR S99 OR S100 OR S101 OR S102 OR S103 OR S104 OR S105 OR S106 OR S107 OR S108 OR S109 OR S110 OR S111 OR S112 OR S113

S113 OR/S2-S43

S112 OR/S2-S43

S111 (MH "Legislation") OR (MH "Jurisprudence")

S110 (MH "Civil Rights") OR (MH "Consumer Advocacy") OR (MH "Patient Advocacy") OR (MH "Patient Rights") OR (MH "Social Justice")

S109 TI (legislat* or jurisprudence or law* or legal*) OR AB (legislat* or jurisprudence or law* or legal*)

S108 TI (civil* or consumer* or patient*) N4 (right* or advoca* or justice) OR AB (civil* or consumer* or patient*) N4 (right* or advoca* or justice)

S107 (MH "Financial Support")

S106 TI (financ* or monetary or economic*) N4 (supp* or assist* or subsid*) OR AB (financ* or monetary or economic*) N4 (supp* or assist* or subsid*)

S105 (MH "Child Care+")

S104 (MH "Home Nursing")

S103 (MH "Caregiver Burden") OR (MH "Caregiver Support") OR (MH "Caregivers")

S102 TI (care giver* or caregiver* or respite or (home* N3 (nurs* or assist* or help*)) or child care or childcare) OR AB (care giver* or caregiver* or respite or (home* N3 (nurs* or assist* or help*)) or child care or childcare)

S101 (MH "Social Isolation+")

S100 (MH "Social Environment") OR (MH "Home Environment")

S99 (MH "Social Capital") OR (MH "Socialization")

S98 (MH "Social Welfare")

S97 (MH "Peer Group") OR (MH "Support Groups")

S96 (MH "Interpersonal Relations")

S95 (MH "Community Networks")

S94 (MH "Discrimination") OR (MH "Discrimination, Employment")

S93 (MH "Social Adjustment") OR (MH "Prejudice")

S92 TI (social* or communit* or famil* or friend* or self help or peer* or colleague* or collegial* or therapeutic) N4 (life* or live* or participat* or adjust* or accept* or distan* or discriminat* or isolat* or relation* or welfare* or integrat* or dispute* or alien* or support* or assist* or group* or club* or organi?ation* or network* or strain* or stress*) OR AB (social* or communit* or famil* or friend* or self help or peer* or colleague* or collegial* or therapeutic) N4 (life* or live* or participat* or adjust* or accept* or distan* or discriminat* or isolat* or relation* or welfare* or integrat* or dispute* or alien* or support* or assist* or group* or club* or organi?ation* or network* or strain* or stress*)

S91 (MH "Community Living+")

S90 TI (walk* or ambulat* or mobil*) N3 (difficult* or limit* or impair*) OR AB (walk* or ambulat* or mobil*) N3 (difficult* or limit* or impair*)

S89 (MH "Assistive Technology Devices+")

S88 TI (assist* or self help) N3 (device* or technolog*) OR AB (assist* or self help) N3 (device* or technolog*)

S87 (MH "Activities of Daily Living+")

S86 TI ("activities of daily living" or adl*) OR AB ("activities of daily living" or adl*)

S85 (MH "Self Administration") OR (MH "Self Medication")

S84 (MH "Self Care")

S83 TI (self or independent or home) N4 (care* or caring or life or lives or living or manag* or medica*) OR AB (self or independent or home) N4 (care* or caring or life or lives or living or manag* or medica*)

S82 (MH "Equipment Design")

S81 (MH "Facility Design and Construction+")

S80 TI (architect* or space* or spacial* or facilit* or residen* or build* or home* or house* or apartment* or dwelling* or flat* or unit* or ramp* or park* or access* or barrier* or design* or product* or object*) OR AB (architect* or space* or spacial* or facilit* or residen* or build* or home* or house* or apartment* or dwelling* or flat* or unit* or ramp* or park* or access* or barrier* or design* or product* or object*)

S79 (MH "Ergonomics+")

S78 TI (ergonomic* or posture*) OR AB (ergonomic* or posture*)

S77 (MH "Transportation+")

S76 TI (transport* or travel*) OR AB (transport* or travel*)

S75 (MH "Job Re-Entry")

S74 (MH "Disability Evaluation+")

S73 (MH "Employment") OR (MH "Employment of Disabled+") OR (MH "Job Security") OR (MH "Job Market") OR (MH "Employment Status")

S72 (MH "Occupational Health") OR (MH "Occupational Hazards") OR (MH "Stress, Occupational")

S71 TI (unemploy* or jobless*) OR AB (unemploy* or jobless*)

S70 TI (work* or employ* or occupation* or job* or industr*) N4 (participat* or retain* or retention or capacity or capability or function* or discriminat* or prejudic* or find* or keep* or gain* or obtain* or secur* or access* or opportunit* or return* or safe* or well* or hygiene or evaluat* or assess* or rehab*) OR AB (work* or employ* or occupation* or job* or industr*) N4 (participat* or retain* or retention or capacity or capability or function* or discriminat* or prejudic* or find* or keep* or gain* or obtain* or secur* or access* or opportunit* or return* or safe* or well* or hygiene or evaluat* or assess* or rehab*)

S69 TI (consumer* OR patient* OR client* OR customer* OR service user*) OR AB (consumer* OR patient* OR client* OR customer* OR service user*)

S68 S55 AND S67

S67 S56 OR S57 OR S58 OR S59 OR S60 OR S61 OR S62 OR S63 OR S64 OR S65 OR S66

S66 TI back ache OR AB back ache

S65 TI sciatic* OR AB sciatic*

S64 TI lumbago OR AB lumbago

S63 TI backache OR AB backache

S62 TI coccyx OR AB coccyx

S61 TI coccydynia OR AB coccydynia

S60 TI dorsalgia OR AB dorsalgia

S59 TI ( (back or lumbar or sciatic*) N4 pain ) OR AB ( (back or lumbar or sciatic*) N4 pain )

S58 TI Spondylosis OR AB Spondylosis

S57 (MH "Spondylosis+")

S56 (MH "Back Pain+") OR (MH "Sciatica")

S55 S1 AND S46 AND S50 AND S54

S54 S51 OR S52 OR S53

S53 (MH "Patient Centered Care")

S52 (MH "Consumer Satisfaction") OR (MH "Patient Satisfaction")

S51 TI (consumer* or patient* or client* or customer* or service user*) N4 (need* or want* or like* or interest* or prefer* or satisf* or perspective* or experience* or attitude* or belief* or practice* or concern* or support* or participat* or advoca* or center* or centr* or orient* or focus* or empower* or expect* or opinion* or view* or perceive* or perception* or tailor* or bespoke or involv* or priorit* or control*) OR AB (consumer* or patient* or client* or customer* or service user*) N4 (need* or want* or like* or interest* or prefer* or satisf* or perspective* or experience* or attitude* or belief* or practice* or concern* or support* or participat* or advoca* or center* or centr* or orient* or focus* or empower* or expect* or opinion* or view* or perceive* or perception* or tailor* or bespoke or involv* or priorit* or control*)

S50 S47 OR S48

S48 (MH "Needs Assessment")

S47 TI (utili* or need* or seek* or retriev* or provid* or provision or source* or aid* or promot* or access* or demand* or insufficien* or deficit* or gap* or barrier* or enabler* or facilitat* or deliver* or implement* or manag* or coordinat*) OR AB (utili* or need* or seek* or retriev* or provid* or provision or source* or aid* or promot* or access* or demand* or insufficien* or deficit* or gap* or barrier* or enabler* or facilitat* or deliver* or implement* or manag* or coordinat*)

S46 S2 OR S3 OR S4 OR S5 OR S6 OR S7 OR S8 OR S9 OR S10 OR S11 OR S12 OR S13 OR S14 OR S15 OR S16 OR S17 OR S18 OR S19 OR S20 OR S21 OR S22 OR S23 OR S24 OR S25 OR S26 OR S27 OR S28 OR S29 OR S30 OR S31 OR S32 OR S33 OR S34 OR S35 OR S36 OR S37 OR S38 OR S39 OR S40 OR S41 OR S42 OR S43 OR S44 OR S45

S45 OR/S2-S43

S44 OR/S2-S43

S43 (MH "Legislation") OR (MH "Jurisprudence")

S42 (MH "Civil Rights") OR (MH "Consumer Advocacy") OR (MH "Patient Advocacy") OR (MH "Patient Rights") OR (MH "Social Justice")

S41 TI (legislat* or jurisprudence or law* or legal*) OR AB (legislat* or jurisprudence or law* or legal*)

S40 TI (civil* or consumer* or patient*) N4 (right* or advoca* or justice) OR AB (civil* or consumer* or patient*) N4 (right* or advoca* or justice)

S39 (MH "Financial Support")

S38 TI (financ* or monetary or economic*) N4 (supp* or assist* or subsid*) OR AB (financ* or monetary or economic*) N4 (supp* or assist* or subsid*)

S37 (MH "Child Care+")

S36 (MH "Home Nursing")

S35 (MH "Caregiver Burden") OR (MH "Caregiver Support") OR (MH "Caregivers")

S34 TI (care giver* or caregiver* or respite or (home* N3 (nurs* or assist* or help*)) or child care or childcare) OR AB (care giver* or caregiver* or respite or (home* N3 (nurs* or assist* or help*)) or child care or childcare)

S33 (MH "Social Isolation+")

S32 (MH "Social Environment") OR (MH "Home Environment")

S31 (MH "Social Capital") OR (MH "Socialization")

S30 (MH "Social Welfare")

S29 (MH "Peer Group") OR (MH "Support Groups")

S28 (MH "Interpersonal Relations")

S27 (MH "Community Networks")

S26 (MH "Discrimination") OR (MH "Discrimination, Employment")

S25 (MH "Social Adjustment") OR (MH "Prejudice")

S24 TI (social* or communit* or famil* or friend* or self help or peer* or colleague* or collegial* or therapeutic) N4 (life* or live* or participat* or adjust* or accept* or distan* or discriminat* or isolat* or relation* or welfare* or integrat* or dispute* or alien* or support* or assist* or group* or club* or organi?ation* or network* or strain* or stress*) OR AB (social* or communit* or famil* or friend* or self help or peer* or colleague* or collegial* or therapeutic) N4 (life* or live* or participat* or adjust* or accept* or distan* or discriminat* or isolat* or relation* or welfare* or integrat* or dispute* or alien* or support* or assist* or group* or club* or organi?ation* or network* or strain* or stress*)

S23 (MH "Community Living+")

S22 TI (walk* or ambulat* or mobil*) N3 (difficult* or limit* or impair*) OR AB (walk* or ambulat* or mobil*) N3 (difficult* or limit* or impair*)

S21 (MH "Assistive Technology Devices+")

S20 TI (assist* or self help) N3 (device* or technolog*) OR AB (assist* or self help) N3 (device* or technolog*)

S19 (MH "Activities of Daily Living+")

S18 TI ("activities of daily living" or adl*) OR AB ("activities of daily living" or adl*)

S17 (MH "Self Administration") OR (MH "Self Medication")

S16 (MH "Self Care")

S15 TI (self or independent or home) N4 (care* or caring or life or lives or living or manag* or medica*) OR AB (self or independent or home) N4 (care* or caring or life or lives or living or manag* or medica*)

S14 (MH "Equipment Design")

S13 (MH "Facility Design and Construction+")

S12 TI (architect* or space* or spacial* or facilit* or residen* or build* or home* or house* or apartment* or dwelling* or flat* or unit* or ramp* or park* or access* or barrier* or design* or product* or object*) OR AB (architect* or space* or spacial* or facilit* or residen* or build* or home* or house* or apartment* or dwelling* or flat* or unit* or ramp* or park* or access* or barrier* or design* or product* or object*)

S11 (MH "Ergonomics+")

S10 TI (ergonomic* or posture*) OR AB (ergonomic* or posture*)

S9 (MH "Transportation+")

S8 TI (transport* or travel*) OR AB (transport* or travel*)

S7 (MH "Job Re-Entry")

S6 (MH "Disability Evaluation+")

S5 (MH "Employment") OR (MH "Employment of Disabled+") OR (MH "Job Security") OR (MH "Job Market") OR (MH "Employment Status")

S4 (MH "Occupational Health") OR (MH "Occupational Hazards") OR (MH "Stress, Occupational")

S3 TI (unemploy* or jobless*) OR AB (unemploy* or jobless*)

S2 TI (work* or employ* or occupation* or job* or industr*) N4 (participat* or retain* or retention or capacity or capability or function* or discriminat* or prejudic* or find* or keep* or gain* or obtain* or secur* or access* or opportunit* or return* or safe* or well* or hygiene or evaluat* or assess* or rehab*) OR AB (work* or employ* or occupation* or job* or industr*) N4 (participat* or retain* or retention or capacity or capability or function* or discriminat* or prejudic* or find* or keep* or gain* or obtain* or secur* or access* or opportunit* or return* or safe* or well* or hygiene or evaluat* or assess* or rehab*)

S1 TI (consumer* OR patient* OR client* OR customer* OR service user*) OR AB (consumer* OR patient* OR client* OR customer* OR service user*)

**S4b: CINAHL Search Strategy: non-healthcare needs and osteoarthritis**

Query Limiters/Expanders

S61 S55 AND S60

S60 S56 OR S57 OR S58 OR S59

S59 TI coxarth* OR AB coxarth*

S58 TI (degen* N4 arth*) OR AB (degen* N4 arth*)

S57 TI osteoarth* OR AB osteoarth*

S56 (MH "Osteoarthritis+")

S55 S1 AND S46 AND S50 AND S54

S54 S51 OR S52 OR S53

S53 (MH "Patient Centered Care")

S52 (MH "Consumer Satisfaction") OR (MH "Patient Satisfaction")

S51 TI (consumer* or patient* or client* or customer* or service user*) N4 (need* or want* or like* or interest* or prefer* or satisf* or perspective* or experience* or attitude* or belief* or practice* or concern* or support* or participat* or advoca* or center* or centr* or orient* or focus* or empower* or expect* or opinion* or view* or perceive* or perception* or tailor* or bespoke or involv* or priorit* or control*) OR AB (consumer* or patient* or client* or customer* or service user*) N4 (need* or want* or like* or interest* or prefer* or satisf* or perspective* or experience* or attitude* or belief* or practice* or concern* or support* or participat* or advoca* or center* or centr* or orient* or focus* or empower* or expect* or opinion* or view* or perceive* or perception* or tailor* or bespoke or involv* or priorit* or control*)

S50 S47 OR S48 OR S49

S49 (MH "Health Services Accessibility") OR (MH "Health Services for Persons with Disabilities") OR (MH "Health Services Needs and Demand")

S48 (MH "Needs Assessment")

S47 TI (utili* or need* or seek* or retriev* or provid* or provision or source* or aid* or promot* or access* or demand* or insufficien* or deficit* or gap* or barrier* or enabler* or facilitat* or deliver* or implement* or manag* or coordinat*) OR AB (utili* or need* or seek* or retriev* or provid* or provision or source* or aid* or promot* or access* or demand* or insufficien* or deficit* or gap* or barrier* or enabler* or facilitat* or deliver* or implement* or manag* or coordinat*)

S46 S2 OR S3 OR S4 OR S5 OR S6 OR S7 OR S8 OR S9 OR S10 OR S11 OR S12 OR S13 OR S14 OR S15 OR S16 OR S17 OR S18 OR S19 OR S20 OR S21 OR S22 OR S23 OR S24 OR S25 OR S26 OR S27 OR S28 OR S29 OR S30 OR S31 OR S32 OR S33 OR S34 OR S35 OR S36 OR S37 OR S38 OR S39 OR S40 OR S41 OR S42 OR S43 OR S44 OR S45

S45 OR/S2-S43

S44 OR/S2-S43

S43 (MH "Legislation") OR (MH "Jurisprudence")

S42 (MH "Civil Rights") OR (MH "Consumer Advocacy") OR (MH "Patient Advocacy") OR (MH "Patient Rights") OR (MH "Social Justice")

S41 TI (legislat* or jurisprudence or law* or legal*) OR AB (legislat* or jurisprudence or law* or legal*)

S40 TI (civil* or consumer* or patient*) N4 (right* or advoca* or justice) OR AB (civil* or consumer* or patient*) N4 (right* or advoca* or justice)

S39 (MH "Financial Support")

S38 TI (financ* or monetary or economic*) N4 (supp* or assist* or subsid*) OR AB (financ* or monetary or economic*) N4 (supp* or assist* or subsid*)

S37 (MH "Child Care+")

S36 (MH "Home Nursing")

S35 (MH "Caregiver Burden") OR (MH "Caregiver Support") OR (MH "Caregivers")

S34 TI (care giver* or caregiver* or respite or (home* N3 (nurs* or assist* or help*)) or child care or childcare) OR AB (care giver* or caregiver* or respite or (home* N3 (nurs* or assist* or help*)) or child care or childcare)

S33 (MH "Social Isolation+")

S32 (MH "Social Environment") OR (MH "Home Environment")

S31 (MH "Social Capital") OR (MH "Socialization")

S30 (MH "Social Welfare")

S29 (MH "Peer Group") OR (MH "Support Groups")

S28 (MH "Interpersonal Relations")

S27 (MH "Community Networks")

S26 (MH "Discrimination") OR (MH "Discrimination, Employment")

S25 (MH "Social Adjustment") OR (MH "Prejudice")

S24 TI (social* or communit* or famil* or friend* or self help or peer* or colleague* or collegial* or therapeutic) N4 (life* or live* or participat* or adjust* or accept* or distan* or discriminat* or isolat* or relation* or welfare* or integrat* or dispute* or alien* or support* or assist* or group* or club* or organi?ation* or network* or strain* or stress*) OR AB (social* or communit* or famil* or friend* or self help or peer* or colleague* or collegial* or therapeutic) N4 (life* or live* or participat* or adjust* or accept* or distan* or discriminat* or isolat* or relation* or welfare* or integrat* or dispute* or alien* or support* or assist* or group* or club* or organi?ation* or network* or strain* or stress*)

S23 (MH "Community Living+")

S22 TI (walk* or ambulat* or mobil*) N3 (difficult* or limit* or impair*) OR AB (walk* or ambulat* or mobil*) N3 (difficult* or limit* or impair*)

S21 (MH "Assistive Technology Devices+")

S20 TI (assist* or self help) N3 (device* or technolog*) OR AB (assist* or self help) N3 (device* or technolog*)

S19 (MH "Activities of Daily Living+")

S18 TI (activities of daily living or adl*) OR AB (activities of daily living or adl*)

S17 (MH "Self Administration") OR (MH "Self Medication")

S16 (MH "Self Care")

S15 TI (self or independent or home) N4 (care* or caring or life or lives or living or manag* or medica*) OR AB (self or independent or home) adj4 (care* or caring or life or lives or living or manag* or medica*)

S14 (MH "Equipment Design")

S13 (MH "Facility Design and Construction+")

S12 TI (architect* or space* or spacial* or facilit* or residen* or build* or home* or house* or apartment* or dwelling* or flat* or unit* or ramp* or park* or access* or barrier* or design* or product* or object*) OR AB (architect* or space* or spacial* or facilit* or residen* or build* or home* or house* or apartment* or dwelling* or flat* or unit* or ramp* or park* or access* or barrier* or design* or product* or object*)

S11 (MH "Ergonomics+")

S10 TI (ergonomic* or posture*) OR AB (ergonomic* or posture*)

S9 (MH "Transportation+")

S8 TI (transport* or travel*) OR AB (transport* or travel*)

S7 (MH "Job Re-Entry")

S6 (MH "Disability Evaluation+")

S5 (MH "Employment") OR (MH "Employment of Disabled+") OR (MH "Job Security") OR (MH "Job Market") OR (MH "Employment Status")

S4 (MH "Occupational Health") OR (MH "Occupational Hazards") OR (MH "Stress, Occupational")

S3 TI (unemploy* or jobless*) OR AB (unemploy* or jobless*)

S2 TI (work* or employ* or occupation* or job* or industr*) N4 (participat* or retain* or retention or capacity or capability or function* or discriminat* or prejudic* or find* or keep* or gain* or obtain* or secur* or access* or opportunit* or return* or safe* or well* or hygiene or evaluat* or assess* or rehab*) OR AB (work* or employ* or occupation* or job* or industr*) N4 (participat* or retain* or retention or capacity or capability or function* or discriminat* or prejudic* or find* or keep* or gain* or obtain* or secur* or access* or opportunit* or return* or safe* or well* or hygiene or evaluat* or assess* or rehab*)

S1 TI (consumer* OR patient* OR client* OR customer* OR service user*) OR AB (consumer* OR patient* OR client* OR customer* OR service user*)

**Table S1: Detailed risk of bias assessments: qualitative Studies**

| **STUDY** | **YEAR** | **Number of participants** | **Study design** | **CASP 1^a^** | **CASP 2 ^a^** | **CASP 3 ^a^** | **CASP 4 ^a^** | **CASP 5 ^a^** | **CASP 6 ^a^** | **CASP 7 ^a^** | **CASP 8 ^a^** | **CASP 9 ^a^** | **CASP 10 ^a^** | **Overall Risk of Bias** |
| --- | --- | --- | --- | --- | --- | --- | --- | --- | --- | --- | --- | --- | --- | --- |
| ***Inflammatory Arthritis Papers*** | | | | | | | | | | | | | | |
| **Hamnes (35)** | 2011 | 16 | Semi-structured interviews | Yes | Yes | Yes | No | Yes | No | Yes | No | Yes | Yes | Moderate |
| **Herrera-Saray (34)** | 2013 | 15 | Semi-structured interviews | Yes | Yes | Yes | No | Yes | No | Yes | No | Yes | Yes | Moderate |
| **Kristiansen (38)** | 2012 | 31 | Focus Groups | Yes | Yes | Yes | Yes | No | No | Yes | No | Yes | Yes | Moderate |
| **Lempp (40)** | 2006 | 26 | Semi-structured interviews | Yes | Yes | Yes | Yes | Yes | No | Yes | Yes | Yes | Yes | Low |
| **Van Der Meer (36)** | 2011 | 14 | Semi-structured interviews | Yes | Yes | Yes | Yes | Yes | No | Yes | No | Yes | Yes | Low |
| **Sverker (41)** | 2014 | 48 | interviews | Yes | Yes | Yes | Yes | Yes | No | Yes | No | Yes | Yes | Low |
| **Bergsten(33)** | 2011 | 16 | Interviews | Yes | Yes | Yes | Yes | Unsure | No | Yes | No | Yes | Yes | Moderate |
| **Been-Dahmen (47)** | 2017 | 20 | Interviews | Yes | Yes | Yes | Yes | Yes | Yes | Yes | Yes | Yes | Yes | Low |
| **Kostova (45)** | 2014 | 20 | Semi-structured interviews | Yes | Yes | Yes | Yes | Yes | No | Yes | Yes | Yes | Yes | Low |
| **Carter** (49) | 2019 | 21 | Semi-structured interviews | Yes | Yes | Yes | Yes | Yes | No | Yes | Unsure | Yes | Yes | Moderate |
| **Thomas** (51) | 2019 | 15 | Semi-structured interviews | Yes | Yes | Yes | Yes | Yes | No | Yes | Yes | Yes | Yes | Low |
| ***Osteoarthritis Papers*** | | | | | | | | | | | | | | |
| **Al-Taiar (29)** | 2013 | 39 | Focus Groups | Yes | Yes | Yes | Yes | Yes | No | Yes | Yes | Yes | Yes | Low |
| **Baumann (31)** | 2007 | 96 | Focus Groups | Yes | Yes | Yes | No | No | No | No | Yes | Yes | Yes | Moderate |
| **Bukhave & Huniche (30)** | 2014 | 31 | Semi-structured interviews | Yes | Yes | Yes | Yes | Yes | No | Yes | No | Yes | Yes | Low |
| **Chan (27)** | 2011 | 20 | Semi-structured interviews | Yes | Yes | Yes | No | Yes | Yes | No | Yes | Yes | Yes | Low |
| **Hill (23)** | 2010 | 29 | Semi-structured interviews | Yes | Yes | Yes | Yes | Yes | No | Yes | Yes | Yes | Yes | Low |
| **Kao (28)** | 2014 | 17 | Semi-structured interviews | Yes | Yes | Yes | No | Yes | No | Yes | Yes | Yes | Yes | Low |

^a^Scored using CASP system (19)

**Table S2: Quantitative Scoring**

| **STUDY** | | **YEAR** | **Number of participants** | **Study design** | **Criteria 1^b^** | **Criteria 2 ^b^** | **Criteria 3 ^b^** | **Criteria 4 ^b^** | **Criteria 5 ^b^** | **Criteria 6 ^b^** | **Criteria 7 ^b^** | **Criteria 8 ^b^** | **Criteria 9 ^b^** | **Criteria 10 ^b^** | **Overall Risk of Bias** |
| --- | --- | --- | --- | --- | --- | --- | --- | --- | --- | --- | --- | --- | --- | --- | --- |
| ***Osteoarthritis Papers*** | | | | | | | | | | | | | | | |
| **Neville (26)** | **1999** | | 197 | Cross-Sectional | No | Yes | No | No | Yes | Yes | No | Yes | No | Yes | High |
| **Ackerman et al (21)** | **2013** | | 126 | Cross-sectional | No | Yes | No | No | Yes | Yes | No | Yes | Yes | Yes | Moderate |
| **Ilori (2016)(22)** | **2016** | | 270 | Cross-sectional | No | Yes | No | Unsure | Yes | Yes | Yes | Yes | Yes | Yes | Moderate |
| **Kjeken (24)** | **2013** | | 125 | Cross-sectional | No | Yes | No | Unsure | Yes | Yes | Yes | Yes | Yes | Yes | Moderate |
| **Tanimura (25)** | **2011** | | 362 | Cross-sectional | No | Yes | No | Unsure | Yes | Yes | Unsure | Yes | Yes | Yes | Moderate |
| **Leung (32)** | 2019 | | 45 | Cross-sectional | No | Yes | No | Unsure | Yes | Yes | Yes | Yes | Yes | Yes | Moderate |
| ***Inflammatory Arthritis papers*** | | | | | | | | | | | | | | | |
| **Henchoz (37)** | | 2013 | 89 | Cross-Sectional | No | Unsure | No | No | Yes | Yes | No | Yes | Yes | Yes | High |
| **Laidmae (39)** | | 2009 | 808 | Cross-Sectional | Yes | Yes | No | No | Yes | Yes | No | Yes | No | Yes | Moderate |
| **Neville (26)** | | 1999 | 197 | Cross-Sectional | No | Yes | No | No | Yes | Yes | No | Yes | No | Yes | High |
| **Giacomelli (42)** | | 2015 | 743 | Cross-sectional | Yes | Yes | No | Yes | Yes | Yes | Unsure | Yes | Yes | Yes | Low |
| **Cunha-Miranda (46)** | | 2010 | 233 | Cross-sectional | No | Yes | No | No | Yes | Yes | No | No | Yes | Yes | High |
| **Sato(48)** | | 2008 | 364 | Cross-sectional | No | Yes | No | No | Yes | Yes | Yes | Yes | Yes | Yes | Moderate |
| **Strand(44)** | | 2015 | 1958 | Cross-sectional | No | Yes | No | No | Yes | Yes | No | No | Yes | Yes | High |
| **Wollenhaupt (43)** | | 2013 | 318 | Cross-sectional | No | Yes | No | No | Yes | Unsure | No | Yes | Yes | Yes | High |
| **Alten** (50) | | 2019 | 1231 | Cross-sectional | No | Yes | No | Unsure | Yes | Yes | No | Yes | Yes | Yes | High |

**Supplementary Material Appendix S1: Hoy’s Risk of Bias Checklist to Assess Bias in Quantitative Studies**

**ASSESSING RISK OF BIAS QUANTITATIVE STUDIES**

1. Was the study’s target population a close representation of the national population in relation to relevant variables?
2. Was the sampling frame a true or close representation of the target population?
3. Was some form of random selection used to select the sample OR was a census undertaken?
4. Was the likelihood of nonresponse bias minimal?
5. Were data collected directly from the subjects (as opposed to a proxy)?
6. Was an acceptable case definition used in the study?
7. Was the study instrument that measured the parameter of interest shown to have validity and reliability?
8. Was the same mode of data collection used for all subjects?
9. Was the length of the shortest prevalence period for the parameter of interest appropriate?
10. Were the numerator(s) and denominator(s) for the parameter of interest appropriate?
